# Association of Aβ with ceramide-enriched astrosomes mediates Aβ neurotoxicity

**DOI:** 10.1186/s40478-020-00931-8

**Published:** 2020-04-28

**Authors:** Ahmed Elsherbini, Alexander S. Kirov, Michael B. Dinkins, Guanghu Wang, Haiyan Qin, Zhihui Zhu, Priyanka Tripathi, Simone M. Crivelli, Erhard Bieberich

**Affiliations:** 1 Department of Physiology, University of Kentucky College of Medicine, 800 Rose Street Room MS519, Lexington, KY 40536 USA; 2 Department of Neuroscience and Regenerative Medicine, Medical College of Georgia at Augusta University, 1120 15th Street, Augusta, 30912 GA USA

**Keywords:** Astrocytes, Exosomes, Mitochondria, Ceramide, Amyloid

## Abstract

Amyloid-β (Aβ) associates with extracellular vesicles termed exosomes. It is not clear whether and how exosomes modulate Aβ neurotoxicity in Alzheimer’s disease (AD). We show here that brain tissue and serum from the transgenic mouse model of familial AD (5xFAD) and serum from AD patients contains ceramide-enriched and astrocyte-derived exosomes (termed astrosomes) that are associated with Aβ. In Neuro-2a cells, primary cultured neurons, and human induced pluripotent stem cell-derived neurons, Aβ-associated astrosomes from 5xFAD mice and AD patient serum were specifically transported to mitochondria, induced mitochondrial clustering, and upregulated the fission protein Drp-1 at a concentration corresponding to 5 femtomoles Aβ/L of medium. Aβ-associated astrosomes, but not wild type or control human serum exosomes, mediated binding of Aβ to voltage-dependent anion channel 1 (VDAC1) and subsequently, activated caspases. Aβ-associated astrosomes induced neurite fragmentation and neuronal cell death, suggesting that association with astrosomes substantially enhances Aβ neurotoxicity in AD and may comprise a novel target for therapy.

## Introduction

Aβ plaque deposits and tau neurofibrillary tangle formation are hallmarks of AD [2, 62]. However, it is still controversial which of the two factors is critical for neuronal dysfunction and death, ultimately leading to cognitive decline and demise of the patient. Most of the previous studies assumed that the buildup of Aβ or tau by themselves induces neurotoxicity [47, 55, 70]. This assumption, however, was in stark contrast to observations in AD mouse models and patients showing significant buildup of plaques and tangles without obvious neuronal cell death [62]. We hypothesized that neurotoxicity of Aβ is mediated by its interaction with an unknown factor. Based on our previous studies showing that Aβ associates with astrocyte-derived exosomes (here termed astrosomes), we tested if this interaction mediates neurotoxicity of Aβ [15, 71].

Exosomes are generated as intraluminal vesicles of multivesicular endosomes and secreted as a type of extracellular vesicles by a large variety of cells and tissues [12, 13, 74]. Exosomes are deemed to serve as carriers for the intercellular transport of micro RNAs and some proteins. Although their size of 100 nm favors a high membrane surface-to-volume ratio, the role of membrane lipids in exosomes remains largely unexplored [18, 21, 67]. Our laboratory discovered that the sphingolipid ceramide is enriched in the membrane of astrosomes [71]. We also showed that ceramide mediates association of Aβ with astrosomes and that this association leads to astrosome aggregation in vitro, a process we suggested to nucleate amyloid plaques in AD brain [15]. However, we do not know if amyloid plaque nucleation is the only or even main function of astrosomes. Recent studies demonstrated that Aβ-associated exosomes cross the blood-brain-barrier and are detectable in serum from AD mice and patients [25, 53, 63]. In fact, exosomes purified from patient serum are proposed as AD biomarkers that are detectable up to a decade prior to clinical symptoms of cognitive decline [25]. While a proportion of serum exosomes is clearly derived from brain, composition and function of these exosomes remains largely unknown.

In the current study using mass spectrometry and anti-ceramide antibody, we found that a proportion of serum-derived serum exosomes is enriched with the same ceramide species previously detected in astrosomes isolated from primary astrocyte culture [15]. We also isolated exosomes from wild type and 5xFAD brain tissue and confirmed the astrocytic origin and Aβ association of tissue and serum-derived exosomes by testing for the presence of the astrocyte marker glial fibrillary acidic protein (GFAP) and Aβ. Aβ-associated astrosomes were taken up by neural cells and specifically transported to mitochondria, thereby inducing mitochondrial damage and caspase activation. Most importantly, the concentration of Aβ associated with astrosomes inducing damage was several orders of magnitude lower than required when using Aβ without astrosomes. Aβ-associated astrosomes induced formation of a pro-apoptotic complex between Aβ and voltage-dependent anion channel 1 (VDAC1), the main ADP/ATP transporter in the outer mitochondrial membrane [50, 65]. These results suggest that astrosomes are the unknown factor mediating neurotoxicity of Aβ by inducing mitochondrial damage and apoptosis. Our data also indicate that Aβ-associated exosomes may comprise a novel pharmacological target for AD therapy.

## Materials and methods

### Cell cultures

The N2a cell line was obtained from ATCC (CCL-131™). The cells were grown to 90% confluence at 37 °C and 5% CO_2_ atmosphere in Dulbecco’s modified Eagle’s medium (DMEM) (Gibco, Invitrogen, CA, USA) supplemented with 10% fetal bovine serum (FBS) on 100 mm plates (Corning, MA, USA). For immunocytochemistry analyses, cells were seeded on poly-L-lysine (Milipore-Sigma, Montana, USA) coated cover slips at 10,000 cells/cover slip. Cells were gradually deprived of serum to allow for differentiation into neuron-like cells. Incubation with exosomes was always performed under serum-free conditions.

Primary neurons were isolated from E16.5-P0 mouse cortices following 30 min trypsinization and trituration with a flame-polished Pasteur pipet. Neurons were plated on polyethylene imine coated T-25 flasks as previously described [15] and maintained 7 days in Neurobasal medium with B27 supplement (Life Technologies) prior to incubation with exosomes. To cultivate human induced pluripotent stem (iPS) cell-derived neuroprogenitor (NP) cells, the ReNcell VM Human NP cell line was obtained from Millipore (Temecula, CA, USA, Cat# SCC008). Cells were maintained according to the supplier’s protocol. Briefly, cells were expanded on laminin-coated 100 mm tissue culture dishes (Corning) in ReNcell NSC maintenance medium (Millipore) supplemented with 20 ng/mL fibroblast growth factor–2 (FGF-2) and 20 ng/mL epidermal growth factor (EGF) (Millipore). The medium was changed daily during the maintenance period. The cells were passaged once a week using Accutase (Millipore). Cells were then differentiated by seeding them at around 60% confluency on freshly laminin-coated dishes and growing overnight in the presence of growth factors, followed by withdrawal of growth factors. The media were replaced every other day up to 10 days during the differentiation period.

### Serum exosome isolation, quantification, and labeling

All experiments using mice were carried out according to an Animal Use Protocol approved by the Institutional Animal Care and Use Committee at University of Kentucky. Sera were isolated from freshly obtained mouse blood. Human exosomes were isolated from sera obtained from the University of Kentucky Alzheimer Disease Center.

**Table:** 

DX	SEX	AGE	BMI	Collection Date	Sample Type
NORMAL	Male	75	27	3/4/2009	Serum
NORMAL	Male	75	25.56	5/7/2009	Serum
NORMAL	Male	75	26.1	11/3/2010	Serum
DEMENTED	Male	74	27.28	2/27/2002	Serum
DEMENTED	Male	75	29.41	5/7/2003	Serum
DEMENTED	Male	74	25	3/23/2005	Serum

Mouse blood was drawn through heart puncture and was allowed to clot at room temperature for 30 min. Blood was then centrifuged at 1800 x g for 10 min at 4 °C. The clear upper layer was transferred to a fresh tube and centrifuged at 3000 x g for 15 min to pellet residual blood cells. Exosomes were extracted using ExoQuick exosome solution (EXOQ; System Biosciences, Inc., Mountain View, CA, USA) according to the manufacturer’s protocol. Briefly, 250 μl aliquots of serum were treated with 67 μl of ExoQuick exosome solution, followed by incubation for 60 min at 4 °C to precipitate total exosomes. Tubes were then centrifuged at 1500 x g for 30 min. Each exosome pellet was resuspended in 100 μl of PBS with 1X Halt™ Protease Inhibitor Cocktail (Thermo Fisher, Massachusetts, USA). In certain experiments exosomes were labeled with PKH67 Green Fluorescent Dye using the Green Fluorescent Cell Linker Kit for General Cell Membrane Labelling (Sigma-Aldrich) according to the manufacturer’s protocol. Briefly, ExoQuick pellets were resuspended in PBS, 1 ml of Diluent C (CGLDIL, Sigma-Aldrich) was then added to each sample. As a control, 1 ml of Diluent C after adding the same volume of PBS was used. Next, 4 μl of PKH67 dye was added to 1 ml of Diluent C then mixed with the exosomes and the control, PKH67/Diluent C mixture was ultra-centrifuged before being added to samples. The samples were allowed to incubate < 5 min on a rotor plate. One ml of 1% BSA was then added to bind excess dye. Samples were ultra-centrifuged at 110,000 xg for 70 min, washed and centrifuged again. For exosome quantification, nanoparticle tracking analysis (NTA) with the ZetaView PMX110 (Particle Metrix) was used. Briefly, exosomes were resuspended in PBS. Two ml of appropriately diluted samples were injected into the ZetaView cell. The instrument was set to obtain NTA measurements at 11 positions, two cycles at each position. During acquisition, temperature was set to 23 °C, camera sensitivity to 82, 30 frames/s, and shutter speed to 250. Polystyrene beads (100 nm) were used for instrument calibration. For exosome incubation with ceramide analogs N-oleoyl serinol (S18 or bis palmitoyl ethanolamine (B16) the exosomes prepared from 5xFAD or control serum were incubated at 37 °C for 16 h with 50 μM S18 or B16.

In addition to the ExoQuick exosome isolation method, we used the Exoeasy Maxi kit (Qiagen, Germany) to isolate exosomes from sera following the manufacturer’s protocol. Briefly, sera were diluted with an equal volume of distilled water to reduce viscosity and they were passed through a 0.45 μm filter to remove larger particles. 1 volume of Exoeasy binding buffer (XBP) was then added to 1 volume of sample. Sample/XBP mix was added onto the Exoeasy spin column and centrifuged at 500 x g for 1 min. Flow-through was discarded and the columns were placed back into the same collection tube. Ten ml Exoeasy washing buffer (XWP) were then added to columns, followed by centrifugation at 500 x g for 5 min to remove residual buffer from the column. Flow-through together with the collection tube were discarded. Spin columns were transferred to fresh collection tubes. Four hundred μl of elution buffer were added to the membrane and incubated for 1 min, followed by centrifugation at 500 x g for 5 min to collect the eluate.

### Brain exosome isolation

This method is a modification to the protocol described by Miltenyi Biotic for isolation and cultivation of astrocytes from adult mouse brain utilizing gentleMACS Octo Dissociator. Briefly, mice were anesthetized using isoflurane inhalation in a chamber followed by perfusion of the whole body with cold 1x PBS to remove blood-derived exosomes from the brain. Mice brains were collected, washed with 1x PBS and cut into eight sagittal slices using sterile scalpel in a petri dish. Brain slices were then transferred to C tubes containing enzymatic dissociation buffer. C tubes were tightly closed and attached upside down onto the sleeves of the gentleMACS Octo Dissociator with Heaters, Program 37C_ABDK_01 being used. Samples were resuspended and applied to a MACS SmartStrainer (70 μm) placed on a 50 mL tube. 10 mL of cold D-PBS were applied onto the MACS SmartStrainer (70 μm). Cell suspensions were centrifuged at 300×g for 10 min at 4 °C, supernatants were carefully transferred to a fresh tube to proceed with exosome isolation. Supernatants were centrifuged at 2000×g for 10 min followed by 10,000×g for 30–40 min then passed through a 0.45 μm filter before following the Exoeasy exosome isolation protocol as described above.

### Immunocytochemistry

N2a, primary cultured neurons, or human neuroprogenitor cells were seeded on poly-L-lysine coated cover slips at a density of 25,000 cells/cover slip. N2a cells were allowed to differentiate by gradual serum deprivation [23]. Two days prior to exosome incubation, exosome-free FBS (EXO-FBS - System Biosciences, Mountain View, CA, USA) was used to supplement the media. Cells were then incubated with exosomes and washed three times with PBS, followed by fixation with 4% p-formaldehyde containing 0.5% glutaraldehyde in PBS for 15 min at room temperature. Permeabilization was performed by incubation with 0.2% Triton X-100 in PBS for 5 min at room temperature. Non-specific binding sites were blocked with 3% ovalbumin/PBS for 1 h at 37 °C. Cells were then incubated with primary antibodies at 4 °C overnight. The next day, cells were washed with PBS and incubated with secondary antibodies diluted 1:300 in 0.1% ovalbumin/PBS for 2 h at 37 °C. Secondary antibodies were Cy2-conjugated donkey anti-mouse IgM, Alexa Fluor 546-conjugated donkey anti-rabbit IgG, and Alexa Fluor 647-conjugated goat anti-mouse IgG (Jackson ImmunoResearch, West Grove, PA). After washing, cover slips were mounted using Fluoroshield supplemented with DAPI (Sigma-Aldrich) to visualize the nuclei. We used the following primary antibodies: anti-ceramide rabbit IgG (1:100, our laboratory), anti-flotillin-2 mouse IgG (1:300 BD Biosciences, California, USA, 610383), anti-amyloid-beta mouse IgG 4G8 clone (1:200 Biolegends, California, USA, SIG-39220), beta amyloid recombinant rabbit monoclonal antibody (H31L21, Thermo Fisher), anti-GFAP mouse IgG (1:500, abcam, Cambridge, MA, USA, ab10062), anti-Tom 20 rabbit IgG (1200, Santa Cruz, sc-11,415), anti-VDAC1 rabbit IgG (1500, Abcam, ab15895). Fluorescence microscopy was performed using Eclipse Ti2-E inverted microscope system (Nikon, New York, USA). Images were processed using Nikon NIS-Elements software equipped with a 3D deconvolution program. Pearson’s correlation coefficient for two fluorescence channels in overlays was used to assess the degree of colocalization.

### Proximity ligation assay

Cells were grown and treated as described above in the protocol for immunocytochemistry. Non-specific binding sites were blocked with Duolink PLA blocking solution (Sigma-Aldrich) for 1 h at 37 °C. The primary antibodies used were; anti-Aβ mouse IgG (1:500 4G8, Biolegends, California, USA, SIG-39220), anti-VDAC1 rabbit IgG (1:1000 abcam, Cambridge, MA, USA, ab34726) Secondary PLA probes: anti-mouse MINUS affinity-purified donkey anti-mouse IgG (H + L) and anti-rabbit PLUS affinity-purified donkey anti-rabbit IgG (H + L) were diluted 1:5 in antibody diluent buffer and samples incubated for 1 h at 37 °C followed by ligation and amplification steps as described in the manufacturer’s protocol (Duolink, Sigma-Aldrich). Cover slips were mounted using Fluoroshield supplemented with DAPI (Sigma-Aldrich) to visualize the nuclei. Images obtained with secondary antibody only were used as negative controls representing the background intensity in a laser channel. ImageJ software (https://imagej.nih.gov/ij/) was used to analyze the pictures. Two channels (DAPI and TRITC) were separated to analyze nuclear staining (DAPI) of the images separately from the TRITC-channel associated with the PLA dots. Firstly, threshold was set in order to identify nucleus and to allow for binary conversion (black and white). Morphological function was used to separate touching nuclei. Nuclei were counted and added to the region of interest (ROI) where the appropriate minimum and maximum pixel area sizes were set. In the other channel, the number of dots (PLA signals) in each cell as identified by labeling of nuclei was calculated with the “Measure” command from the ROI manager using single point as an output type.

### Isolation of mitochondria

N2a cells were seeded on 100 mm dishes at 35–40% of density, followed by incubation with wild type or 5xFAD serum exosomes. Sixteen hours later, cells were harvested and washed twice with ice-cold PBS. Cell pellets were then transferred into a Dounce homogenizer and disrupted with 2 ml of ice-cold mitochondria extraction buffer [10 mM HEPES, 125 mM sucrose, 0.01% BSA, 250 mM mannitol, 10 mM EGTA, and protease inhibitors (pH 7.2)]. The homogenates were transferred into a centrifuge tube and cell debris pelleted at 700 x g at 4 °C for 10 min to enrich for mitochondria. Following centrifugation under same conditions, supernatants were transferred to a new ice-cold tube, and then mitochondria pelleted at 10,000 x g for 15 min at 4 °C. The mitochondrial pellet was resuspended in 1 ml of lipid binding buffer [20 mM Tris-HCl, 150 mM NaCl, 1 mM EDTA (pH 7.5), and 1% digitonin, supplemented with protein inhibitor cocktail (Roche)]. Complete lysis of mitochondrial membranes was achieved by sonication. Removal of insoluble debris was achieved by centrifugation at 10,000 x g for 15 min at 4 °C. The protein concentration in the supernatants from untreated cells and treated cells was determined using Bio-rad RC DC™ Protein Assay.

### FLICA and cytotoxicity assay

The FLICA 660 Poly Caspase Assay Kit (ImmunoChemistry Technologies, Minnesota, USA) was used to determine the presence of early caspase activation. This in vitro assay employs the fluorescent inhibitor probe 660-VAD-FMK to label active caspase enzymes in living cells. N2a cells (0.25–1·10^5^) were incubated with exosomes (0.5–1·10^4^ exosomes/cell) for 6 h at 37 °C. The cells were washed twice with PBS and resuspended in RPMI medium with 10% FBS before staining with 30 × FAM-VAD-FMK for 30 min at 37 °C. Cells were washed with 1 x apoptosis wash buffer prior to being fixed with 4% paraformaldehyde supplemented with 0.5% glutaraldehyde. The assay was then followed by PLA as described above.

For LDH cytotoxicity assays, N2a cells were seeded at a density of 5000 cells/well on 96-well plates in complete culture medium and were allowed to grow to adequate confluency. One day before incubation with exosomes, media were replaced with 2% EV-depleted FBS and kept overnight. Cells were treated for 12 h with 10^4^ exosomes/cell. LDH release was detected using the CyQUANT™ LDH Cytotoxicity Assay (Thermo Fisher Scientific, Waltham, MA, USA) according to the manufacturer’s protocol.

### Western blot and dot blot

For Western blot analysis, samples were mixed with an equal volume of 2X Laemmli sample buffer. Samples were resolved by SDS gel electrophoresis on polyacrylamide gels and transferred to nitrocellulose membrane (Hybond ECL, Amersham Biosciences, UK). Non-specific binding sites were blocked with 5% fat-free dry milk in PBS containing 0.05% Tween-20 followed by overnight incubation with primary antibodies. For exosome characterization we used CD9, CD63, CD81 rabbit antibodies from ExoAb Antibody Kit (System Biosciences, Inc., Mountain View, CA, USA) after dilution to 1:1000. The following primary antibodies were used for immmunolabeling on Western blots: anti-flotillin-2 mouse IgG (1:1000, BD Biosciences, California, USA, 610383), anti-cleaved caspase-3 rabbit IgG (Cell Signaling, Danvers, MA, USA, #9664), anti-VDAC1 goat polyclonal IgG (1: 200, Santa Cruz Biotechnology, Inc., CA, USA), anti-Drp-1 mouse IgG1 kappa light chain (Santa Cruz, Dallas, TX, USA, sc-271,583). Signals were detected using either pico or femto chemiluminescent (ECL) horseradish peroxidase (HRP) substrate (Thermo Fisher, Massachusetts, USA). Blot images were developed using Azure c600 system (Azure Biosystems, California, USA).

### Exosome immune capturing on beads: affinity purification using ceramide beads

Twenty μL of protein A sepharose conjugated magnetic beads were pre-blocked with FcR Blocking Reagent (MACS, Miltenyi Biotec) for 1 h at room temperature. After 3-times washing with lipid biding buffer [20 mM Tris-HCl, 150 mM NaCl, 1 mM EDTA (pH 7.5)], either anti-ceramide rabbit IgG or control non-specific rabbit IgG were immobilized on the beads in 1% BSA. Approximately 2 μg were added to each sample and the reaction kept mixing overnight on a rotary plate. Next day, beads were washed 3-times and diluted exosome samples were added and allowed to incubate with the beads for 2 h at room temperature. Beads were then collected using magnetic columns and washed 3-times with detergent free lipid binding buffer. The beads were incubated with an adequate volume of 2x sample Laemmli buffer, heated at 90 °C for 10 min and processed for immunoblot labeling of GFAP. Aliquots of the flow through were used for dot blots determing Aβ content and the residual sample processed for Western blot using 4 x sample Laemmli buffer. Equal volumes of the samples were then applied to each well for Western blot analysis. 4 μL were used for dot blot with the flow through of each sample.

### Mass spectrometric analysis of lipids

Exosomes prepared from serum were taken up in water and ceramide species were quantified in the sphingolipidomics (LC-MS/MS) analysis core facility at the Medical University of South Carolina, Charleston, SC. The lipid concentration was normalized to lipid phosphate and exosome number.

### Statistical analysis

Clustering analyses were performed with Particle Explorer V2.1.4 (Particle Metrix Inc., Germany) using the following features (1. Particle size 2. Position 3. Area std. 4. Mean intensity std. 5. Trajectory total distance, std. speed, track time, med- speed, and max-speed). For the lipid analysis, results were analyzed with Two-way ANOVA using ceramide species and genetic background as two independent factors. The effect of exosomes from two sources (e.g., wild type and 5xFAD) with unequal sample sizes or unknown variances were analyzed by unpaired t-test with Welch’s correction. When multiple comparisons affected by a potential baseline shift in each sample (e.g., mass spectrometric analysis of ceramide species) were analyzed, we used the Bonferroni correction on One-way ANOVA, a statistical test typically applied to mass spectrometric analyses to exclude false positives. Other tests such as One-way ANOVA with Student-Newman-Keuls (SNK) post hoc test or Tukey correction for comparison of multiple means were applied when used for similar analyses as described in literature. Results showing *p* < 0.05 were reported as statistically significant. All statistical analysis were done on Graphpad prism software.

## Results

### 5xFAD mouse and AD patient serum contains exosomes enriched with ceramide and derived from astrocytes (astrosomes)

**Fig. 1:**
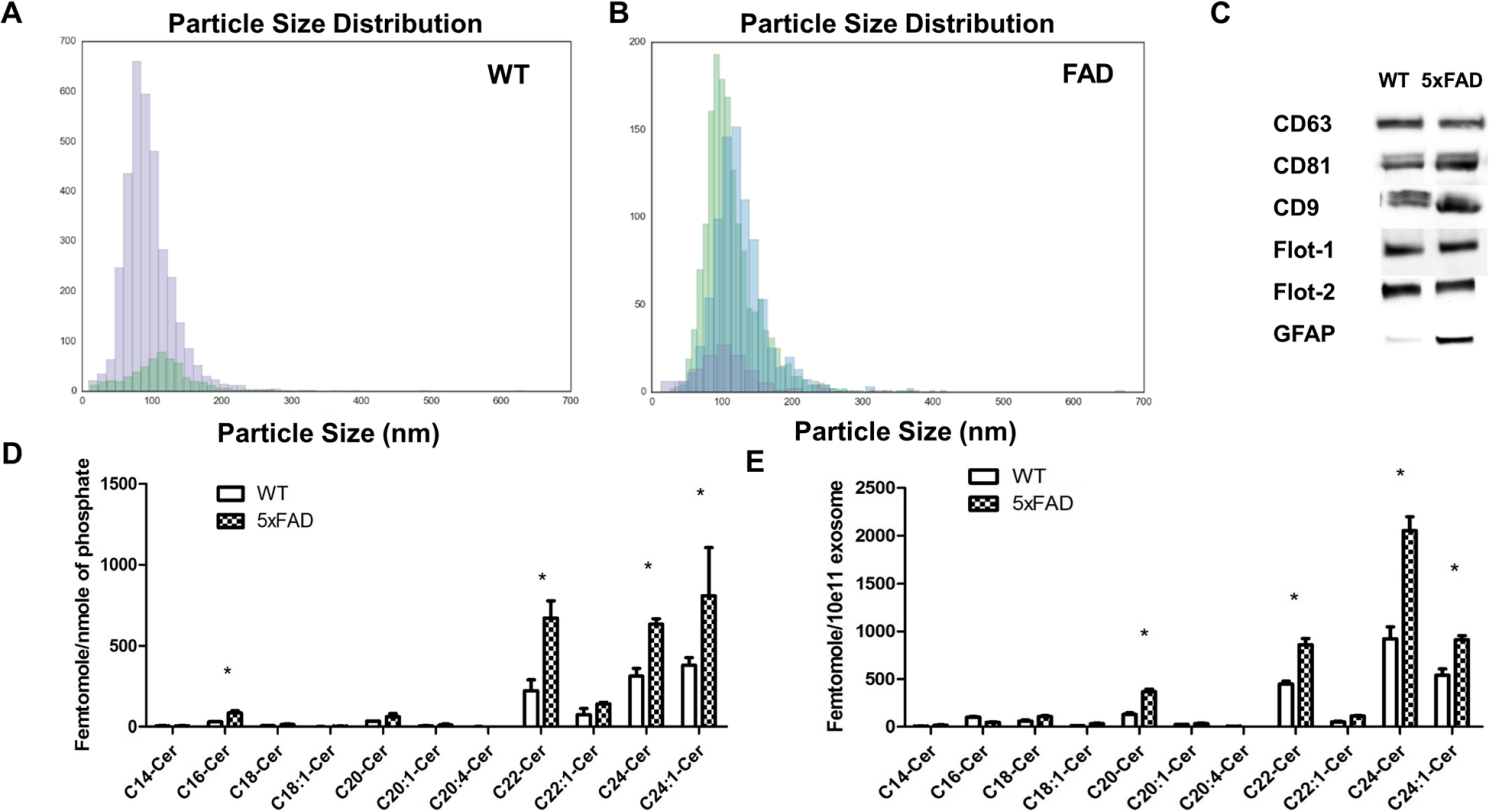
*5xFAD serum-derived exosomes are enriched with ceramide and associated with GFAP*. **a-b** Cluster analysis of wild type (WT) and 5xFAD serum-derived exosomes after Nano Particle Tracking analysis showing a population of larger exosomes in 5xFAD serum. *N* = 3 (**c**) Immunoblot of exosome markers CD9, CD63, CD81, flotillin-1, and flotillin-2, demonstrating higher amounts of GFAP in 5xFAD exosomes compared to WT exosomes. **d-e** Ceramide species profile determined using LC-MS/MS of WT and 5xFAD serum-derived exosomes and normalized to lipid phosphate content (**d**) and to exosome count (**e**) Asterisks denote significance (*p* < 0.05) after Two-way ANOVA followed by Bonferroni correction (*N* = 3)

Several studies showed that exosomes cross the blood-brain-barrier (BBB) carrying toxic and misfolded protein of CNS origin [25, 64]. These studies also showed that purification of exosomes from serum or plasma allows characterization of exosomes from different cell types in the brain, including astrocytes. We used polymer precipitation and membrane affinity chromatography to isolate exosomes from sera of transgenic mouse model of AD and AD patients as these isolation methods were shown to give consistent results when used with plasma or serum [22, 32, 69]. Due to the limitations in availability of AD patient serum, we first focused on characterization of exosomes prepared from serum of the transgenic mouse model of AD (5xFAD) and wild type littermates with identical genetic background (C57Bl/6). 5xFAD mice overexpress presenilins (PS1) with two FAD mutations (M146L and L286V) as well as amyloid precursor protein (APP) with three FAD mutations (V717I, I716V, and K670N/M671L) [49]. Nanoparticle tracker analyses (NTA, Zetaview) and cluster analyses software (Particle Explorer, Particle Metrix, Mebane, NC) showed that the number of exosomes in wild type and 5xFAD serum from 9 months old mice was similar (8.47·10^11^ +/− 3.6·10^10^ exosomes/250 μl serum vs. 9.14·10^11^ +/− 5.1 10^10^ exosomes/250 μl serum, *N* = 6). While the majority of exosomes from wild type serum was composed of a homogenous population of vesicles with medium size of 100 nm (Fig. 1a), exosomes from 5xFAD serum contained an additional vesicle population of larger size accounting for 37 +/- 4% of the total population, indicating aggregate formation (Fig. 1b). Immunoblot analysis was used to validate the presence of exosomal markers such as tetraspanin proteins (CD63, C9, and CD81) as well as raft and exosome-associated proteins flotillin-1 and flotillin-2, and the astrocyte marker GFAP (Fig. 1c).

Lipid analysis using mass spectrometry (LC-MS/MS) showed that 5xFAD exosomes were enriched with ceramide (4.3 pmoles total ceramide/10^11^ 5xFAD serum exosomes vs. 2.2 pmoles/10^11^ wild type serum exosomes), particularly C16:0, C18:0, C20:0, 22:0, C24:0, and C24:1 ceramide (Fig. 1d and e, *N* = 3). Normalization to lipid phosphate (Fig. 1d) as well as particle count (Fig. 1e) showed similar enrichment, confirming that the ceramide composition was representative for the exosome population in serum. We also determined ceramide composition and GFAP association of exosomes in serum from AD patients. Consistent with the results obtained with mouse serum, the number of exosomes in serum from healthy controls and AD patients was similar (2.05·10^11^ +/− 5.1·10^9^ exosomes/250 μl serum vs. 1.85·10^11^ +/− 6.2·10^9^ exosomes/250 μl serum. *N* = 3). There was a population of larger particles which appeared to be similar to that in 5xFAD serum (Supplemental Fig. 1A). Supplemental Fig. 1b shows that the levels of some of the ceramide species (C16:0, C18:0, C18:1, C20:0, and C20:1 ceramide) were increased in AD patient exosomes, while others (C22:0 and C24:0 ceramide) were not. This difference in the ceramide profiles between the 5xFAD mouse and AD patient serum exosomes could be due to differences in the activity of ceramide synthases (CerS) in mice vs. patients. The GFAP level associated with serum exosomes from AD patients was comparable to that of healthy controls (Supplemental Fig. 1C). It is possible that characteristics such as exosome enrichment with GFAP are more profound with 5xFAD serum exosomes because of the severe AD pathology phenotype that may not be completely comparable to that of late onset AD patients. Therefore, our results suggest that the main difference between 5xFAD and AD serum exosomes to those from wild type and human controls is a proportion of exosomes enriched with particular ceramides.

### Serum astrosomes are associated with Aβ and sensitive to novel ceramide analogs

**Fig. 2:**
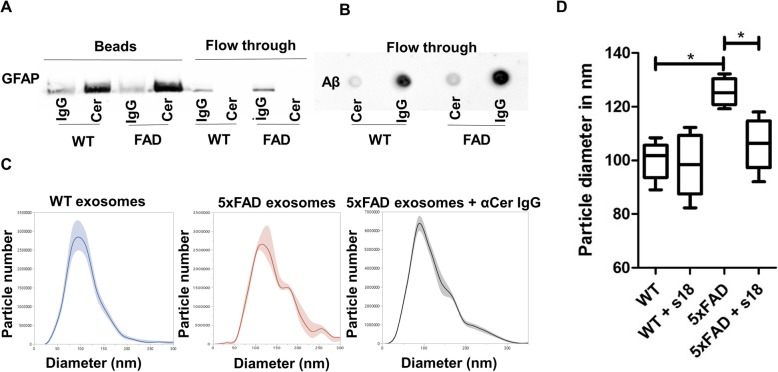
*5xFAD serum astrosomes associated with Aβ form aggregates, which is reduced by the novel ceramide analog S18.***a** Gel electrophoresis after immune capturing of exosomes on beads using either ceramide antibody or control IgG and probing with anti-GFAP antibody. Blot is representative of the results from three independent experiments. **b** Dot blot against Aβ using flow through for the same experiment. **c** Size distribution of wild type (WT), 5xFAD, and 5xFAD exosomes treated with anti-ceramide IgG. **d** Size distribution of WT, 5xFAD, and 5xFAD exosomes treated with the novel ceramide analog S18. Particle diameter of each sample is represented as ±SEM, two-way ANOVA, *p < 0.05. *N* = 4

To further characterize the proportion of ceramide-enriched exosomes, we used anti-ceramide rabbit IgG immobilized on protein A sepharose beads to separate ceramide-enriched exosomes from other exosome populations in serum. Figure 2a shows that GFAP labeling was only found with exosomes bound to the beads, while exosomes in the flow through were GFAP negative. Control rabbit IgG did not bind any serum-derived exosomes confirming specificity of the binding reaction for ceramide-enriched astrosomes. Wild type serum also contained astrosomes retained by anti-ceramide antibody, however, at lower concentration as indicated by weaker immunolabeling for GFAP. NTA analysis showed that retention by anti-ceramide beads reduced the number of exosomes by 2.3+/− 0.3% from wild type and 9.2+/− 0.8% (*N* = 3) from 5xFAD serum indicating that the proportion of ceramide-enriched astrosomes in 5xFAD serum is ≃ 4-fold higher than that in wild type serum.

Next, we tested if ceramide-enriched exosomes in serum were associated with Aβ by determining the amount of Aβ retained on anti-ceramide beads vs. that in flow through. Immunolabeling using dot blots showed that only the flow through of beads with control IgG contained Aβ_42_, while amyloid peptide was retained on anti-ceramide beads (Fig. 2b). Consistent with immunolabeling for GFAP, the amount of Aβ was 2.2-fold higher in 5xFAD serum than that from wild type mice (not shown). Since ceramide-enriched exosomes were associated with GFAP as well as Aβ we concluded that 5xFAD serum contained a proportion of astrosomes enriched with ceramide and associated with Aβ.

Enrichment of astrosomes with ceramide suggested that this lipid participates in association of Aβ to astrosomes. This hypothesis is consistent with our previous studies showing that anti-ceramide IgG prevented aggregation of exosomes induced by incubation with Aβ [15]. Figure 2c shows that incubation with anti-ceramide IgG abolished the proportion of larger sized vesicles in the preparation of 5xFAD exosomes, similar to the effect of anti-ceramide antibody on aggregation of Aβ-associated astrosomes derived from cell culture media. We also found reduction of vesicle size by 17% (*N* = 4) when adding the novel ceramide analog N-oleoyl serinol (S18) but not N-palmitoyl bisethanolamine (B16, structures are shown in Supplemental Fig. 1 D) to 5xFAD exosomes, suggesting that S18 is a ceramide mimic that disrupts Aβ association and aggregation of astrosomes, probably by interfering with the ceramide-mediated binding of Aβ to astrosomes.

### Astrosomes are taken up by neural cells and transport Aβ and ceramide to mitochondria

**Fig. 3:**
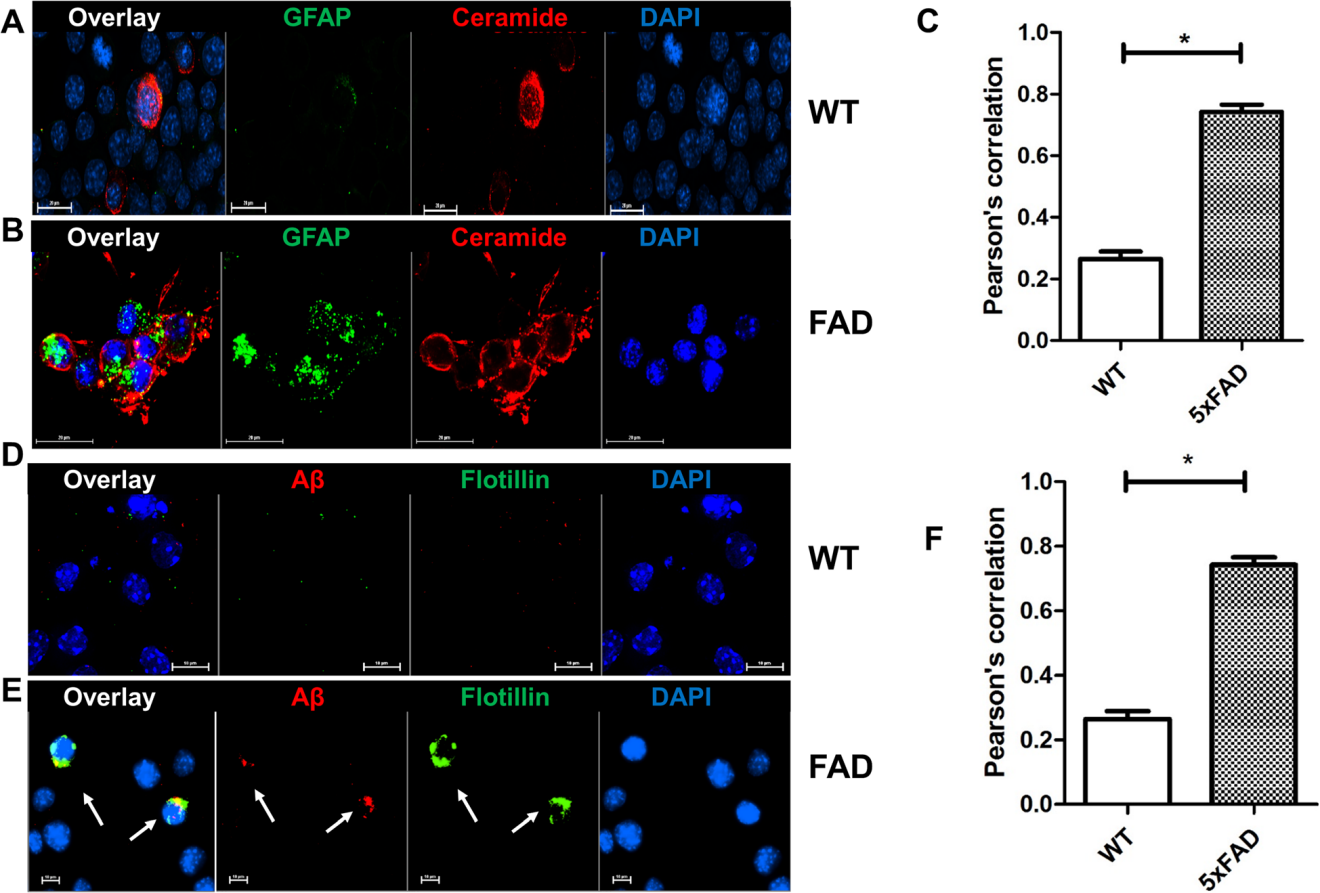
*5xFAD serum contains Aβ-associated astrosomes that are taken up by neural cells* Representative images of N2a cells incubated with exosomes isolated from wild type (WT) (**a**, **d**) or 5xFAD serum (**b**, **e**)and coimmunolabeled with antibodies against GFAP and ceramide (**a**, **b**) or flotillin-2 and Aβ (**d**, **e**). Arrows point at cells with uptake of Aβ-associated exosomes. The Pearson’s correlation coefficient was calculated to compare colocalization of GFAP and ceramide (**c**) or flotillin-2 and Aβ signals (**f**) in WT (open bar) and 5xFAD (closed bar). Welch’s *t*-test, *p < 0.05. *N* = 6

To test if serum-derived exosomes are up taken by neural cells, we incubated neuronally differentiated N2a cells, a mouse neuroblastoma cell line, with exosomes labeled with the fluorescent membrane-binding dye PKH67. N2a cells were used as an in vitro model since neuroblastoma cells are an established model for neuronal uptake and biological activity of exosomes [33, 36]. Key results were then confirmed using primary cultured neurons. Supplemental Fig. 2A-C shows that both, wild type and 5xFAD serum-derived exosomes labeled with PKH67 are taken up by N2a cells and primary cultured neurons. Cells incubated with wild type serum-derived exosomes were labeled for ceramide, but not or only weakly for GFAP (Fig. 3a and c), while cells incubated with 5xFAD serum exosomes were colabeled for ceramide and GFAP (Fig. 3b and c). Since there were no or only few cells that showed increased ceramide signals without being colabeled for GFAP, our data demonstrate that N2a cells effectively take up ceramide-enriched astrosomes.

Next, we tested whether astrosomes transported Aβ into N2a cells. Using immunocytochemistry, we detected Aβ signals in N2a cells incubated with 5xFAD exosomes but not with those from wild type serum (Fig. 3d and e). The Aβ signal colocalized with labeling for flotillin-2 (arrows in Fig. 3e), suggesting that astrosomes delivered Aβ into N2a cells. To further confirm the validity of these results, we used a proximity ligation assay (PLA) for complex formation between ceramide and Aβ in membrane dye PKH67-labeled exosomes taken up by N2a cells [35, 37]. Supplemental Fig. 3A-D shows that PLA signals colocalized with PKH67 labeling and were only observed in cells incubated with 5xFAD exosomes.

**Fig. 4:**
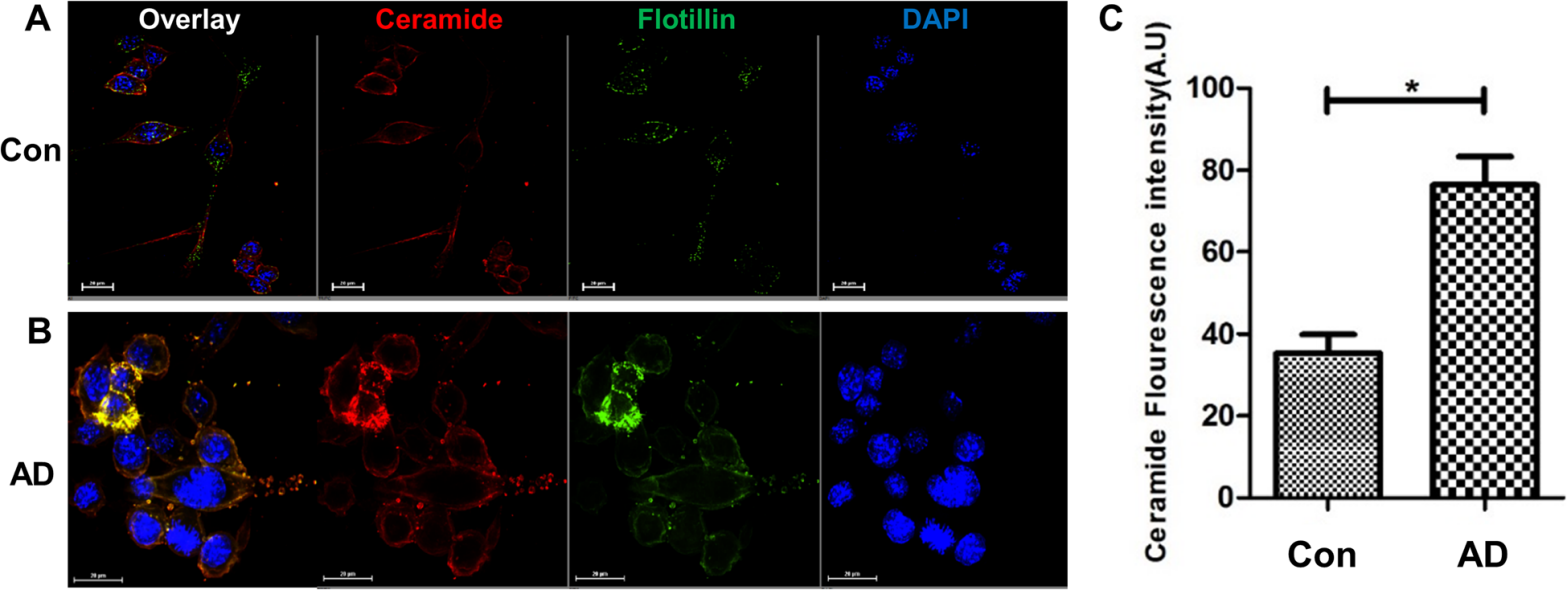
*Serum-derived exosomes from AD patients transport ceramide into cells.* Immunofluorescence images of N2a cells incubated with either (**a**) healthy control or (**b**) AD patient serum-derived exosomes labeled with anti-ceramide and flotillin-2 antibodies. (**c**) fluorescence intensities for the ceramide signal. *N* = 6. Student t-test with Welch’s correction, *p < 0.05, **p < 0.01

We then tested if serum exosomes from AD patients showed similar uptake characteristics as 5xFAD exosomes. N2a cells incubated with AD patient exosomes showed colocalization of ceramide and flotillin-2 (Fig. 4b). While endogenous ceramide and flotillin-2 were detectable in exosome-treated cells, we lowered the pertinent fluorescence signals to that of background to specifically monitor ceramide and flotillin-2 contributed by exosomes. Cells incubated with exosomes from healthy controls showed ceramide and flotillin-2 and ceramide labeling, however, at much lower intensity than cells incubated with AD exosomes (Fig. 4a). These results indicated that exosomes from human serum, particularly when derived from AD patients, were taken up and transported ceramide into cells.

**Fig. 5:**
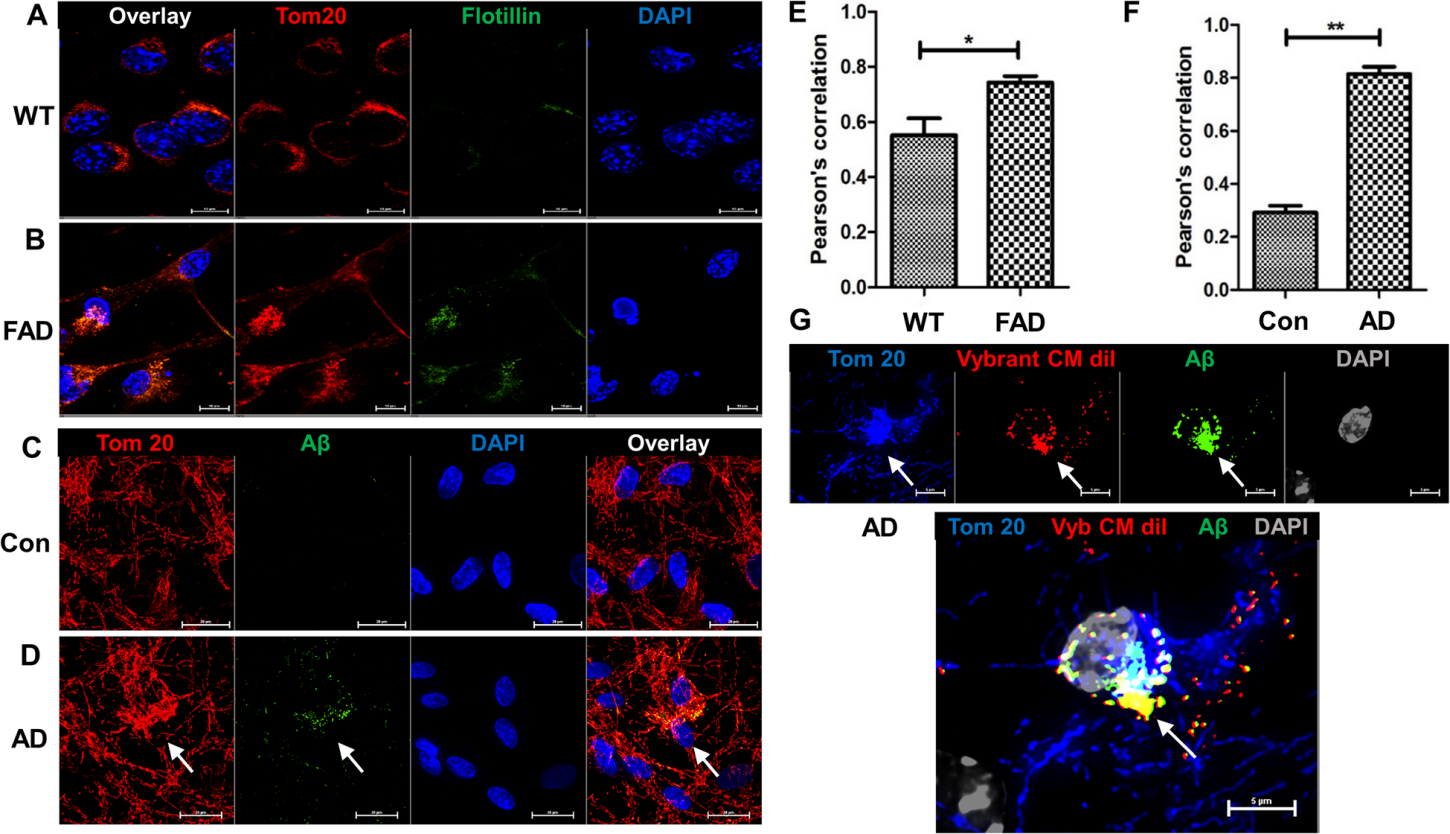
*Serum-derived exosomes from 5xFAD mice and AD patients shuttle Aβ to mitochondria in N2a cells and neurons.* Immunofluorescence images of N2a cells incubated with either (**a**) wild type or (**b**) 5xFAD serum-derived exosomes and then labeled with flotillin-2 and Tom-20 antibodies. (**e**) Pearson’s correlation coefficient for colocalization of flotillin-2 and Tom-20. *N* = 6. Student t-test with Welch’s correction. *p < 0.05. **c, d** Neurons differentiated from human iPS cells and incubated with control healthy human (**c**) or AD patient exosomes (**d**) showing that only AD patient exosomes shuttle Aβ to mitochondria in neurons (arrows). **f** Pearson’s correlation coefficient for colocalization of Aβ with Tom-20. *N* = 6. Student t-test with Welch’s correction. **p < 0.01. **g** as in **d**, but additional labeling of AD patient exosomes with Vybrant CM diI showing mitochondrial clustering (arrows) induced by Aβ-associated exosomes. Bottom image shows detail of (**g**)

Several studies showed that mitochondria are affected by Aβ [9, 45, 57]. Using immunocytochemistry for Aβ and Tom-20, we showed that Aβ was labeled in mitochondria of hippocampal tissue from AD patients (arrows in Supplemental Fig. 4A), suggesting that Aβ is transported to mitochondria in AD brain. To investigate exosome-mediated transport of Aβ, we first tested if 5xFAD serum-derived exosomes are transported to mitochondria. Figure 5a, b and e shows that the exosome marker flotillin-2 colocalized with the mitochondrion marker Tom-20 in 5xFAD exosome-incubated N2a cells. The flotillin fluorescence signal was analyzed after subtracting intrinsic signals, thereby eliminating the possibility that the colocalization resulted from the fluorescence signal of endogenous flotillin with mitochondria. Colabeling and Pearson’s coefficient for colocalization were significantly, but only moderately (about 20%) lower when exosomes from wild type serum were used (Fig. 5b and e), suggesting that transport of exosomes to mitochondria is not critically dependent on Aβ association.

We then tested if serum-derived exosomes from AD patients and healthy controls matched for sex, age, and body matrix index (BMI) transported Aβ to mitochondria in neurons differentiated from human iPS cells. Figure 5d and f shows that Aβ colocalized with Tom-20 in AD patient exosome-treated N2a cells, while there was no Aβ signal detectable when cells were incubated with serum exosomes from healthy controls (Fig. 5c and f). AD patient exosomes labeled with Vybrant CM diI also colocalized with Aβ and Tom-20, demonstrating that exosomes effectively transported Aβ to mitochondria (Fig. 5g). Mitochondria appeared to be clustered, suggesting that uptake of AD patient-derived exosomes led to mitochondrial damage in neurons.

### Astrosomes induce Aβ-VDAC1 complex formation, which activates caspases

**Fig. 6:**
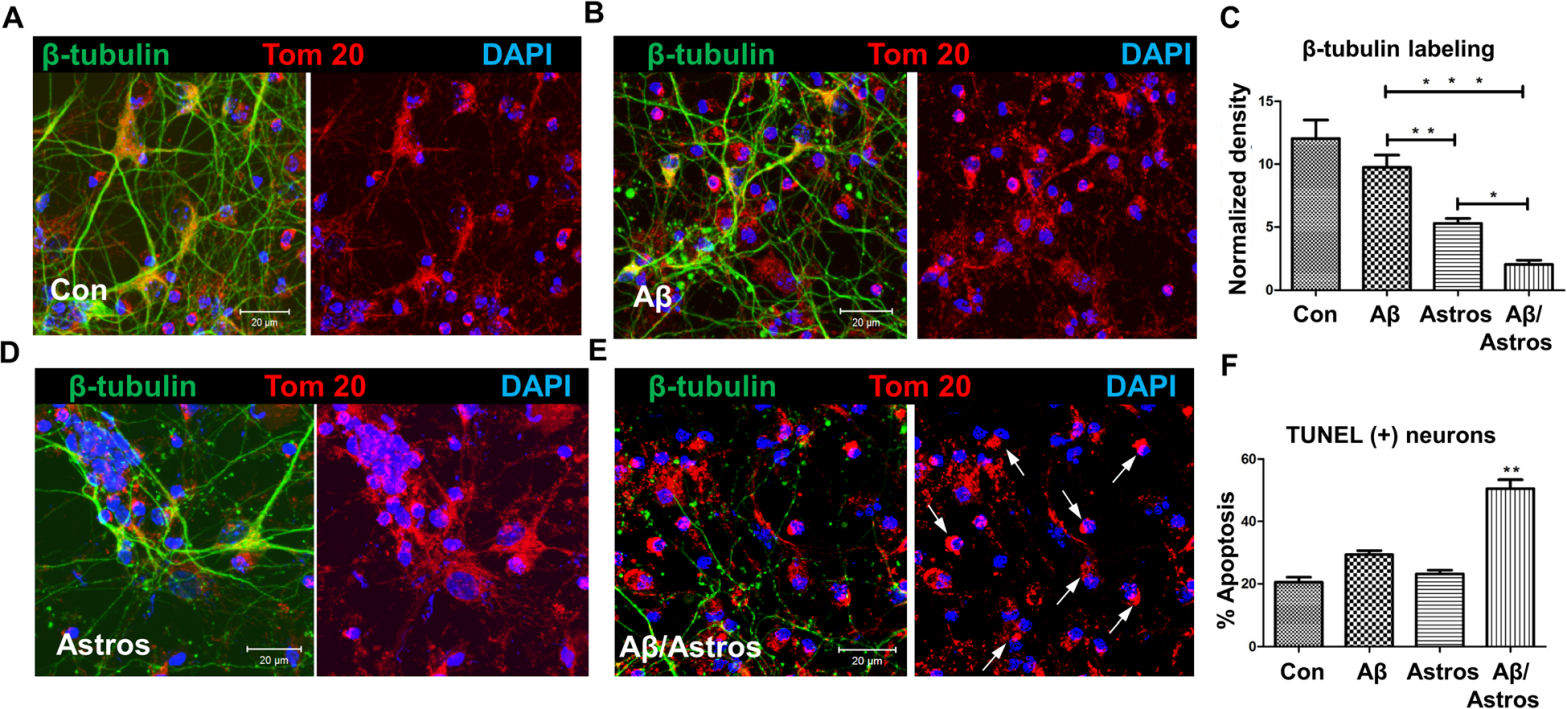
*Neurotoxic effect of Aβ*_*42*_*/astrosome complexes on primary neuronal cultures.* Representative single-focal-plane images of β-tubulin and Tom-20 labeling obtained with control (**a**), Aβ_42_ (**b**), astrosome (**d**), or Aβ_42_/astrosome-incubated (**e**) primary cultured mouse neuron. Arrows indicate mitochondrial clusters. **c** Average normalized density of β-tubulin labeling reveals that the greatest loss occurs in cultures treated with Aβ_42_/astrosome complexes. *N* = 6. One-way ANOVA with Student-Newman-Keuls (SNK) post hoc test). ***p < 0.001, **p < 0.005, *p < 0.05). **f** TUNEL assay detected a 2.6-fold increase in neuronal cell death when Aβ_42_ and astrosomes were combined (*N* = 4. One-way ANOVA with SNK post hoc test)

Our observation that 5xFAD mouse and AD patient serum exosomes induced clustering of mitochondria in N2a cells and primary cultured neurons prompted us to investigate if Aβ-associated astrosomes are neurotoxic by inducing mitochondrial damage. To test if astrosomes themselves were neurotoxic we analyzed mitochondrial clustering and fragmentation of neuronal processes, and performed TUNEL assays after incubation of primary cultured neurons from mouse brain with astrosomes, Aβ, and Aβ pre-incubated with astrosomes (Fig. 6a-f). The number of TUNEL positive cells was increased by 2.6-fold (Fig. 6f) when cells were incubated with Aβ-associated astrosomes, concurrent with mitochondrial clustering (arrows in Fig. 6e) and 5.9-fold enhanced fragmentation of neuronal processes (Fig. 6c) as determined by β-tubulin labeling. This result showed that astrosomes themselves were only marginally toxic, but they significantly enhanced neurotoxicity of Aβ.

**Fig. 7:**
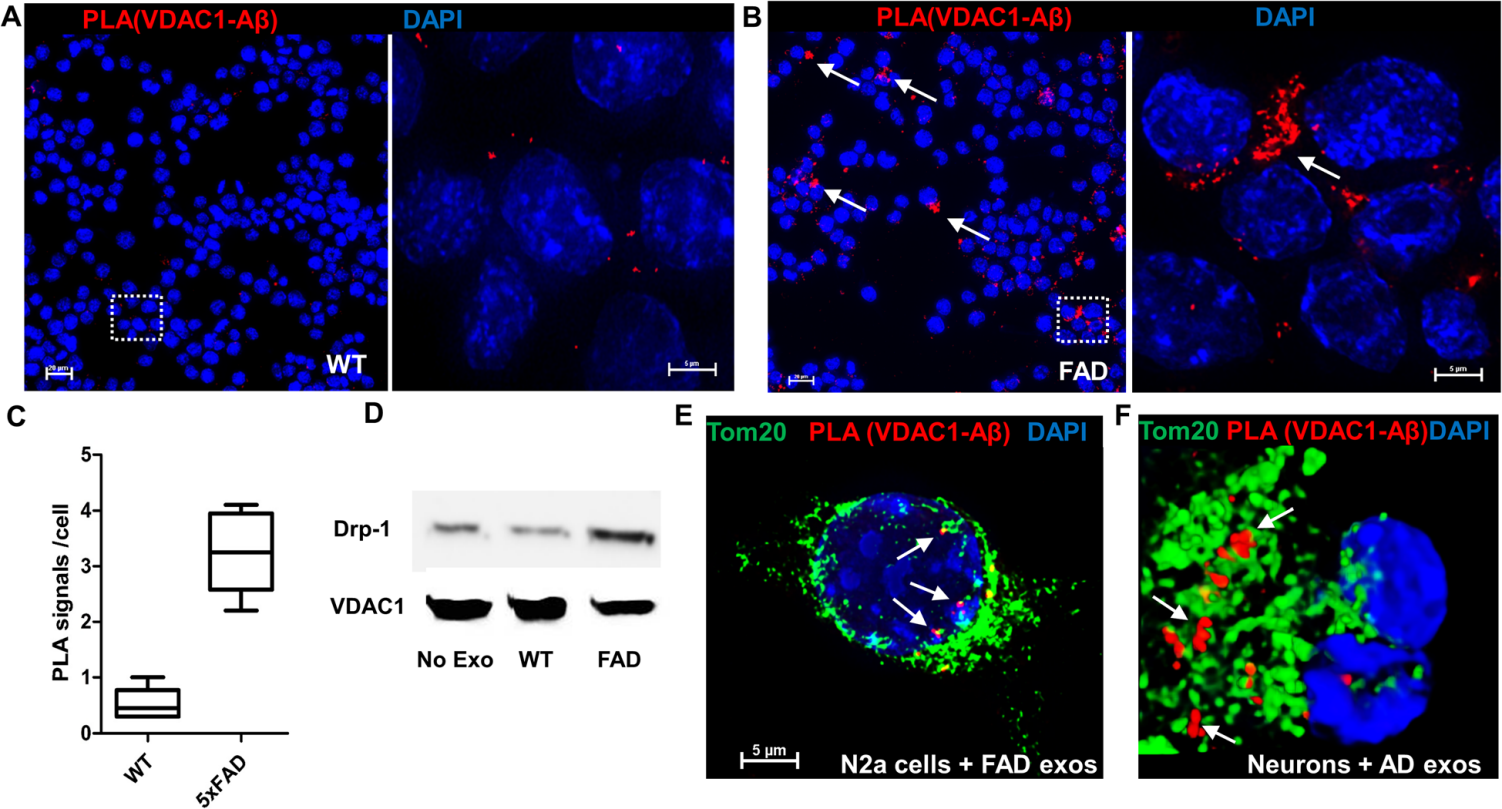
*Aβ-associated exosomes mediate complex formation between VDAC1 and Aβ and induce mitochondrial damage*. **a, b** Representative immunofluorescence images of N2a cells incubated with wild type exosomes (**a**) or 5xFAD exosomes (**b**) showing increased number of PLA signals in cells incubated with 5xFAD exosomes. Right panels show detail from left panel (frame). Each red dot denotes complex formation between Aβ and mitochondrial VDAC1. Arrows indicate PLA signals in mitochondrial clusters (**c**) Calculation and comparison of average PLA signals per cell between wild type and 5xFAD incubations, six images for each condition. Student t-test followed by Welch’s correction. *N* = 6. *p* < 0.001 (**d**) Western blot of mitochondrial protein from N2a cells using antibody against Drp-1 and VDAC1 as a reference protein. **e, f** Colocalization between PLA signals for VDAC1-Aβ complexes and mitochondrial marker Tom-20 labeling in N2a cells and primary cultured neurons incubated with 5xFAD serum exosomes (**e**) and AD patient serum exosomes (**f**)

Mitochondrial dysfunction is known to be a critical factor in induction of neurotoxicity leading to neurodegeneration in AD [11, 19, 51]. One of the previously described targets for Aβ is mitochondrial voltage-dependent anion channel 1 (VDAC1), a mitochondrial gatekeeper for ADP/ATP and calcium localized in the outer mitochondrial membrane [50, 65]. We tested if astrosome-associated Aβ interacted with VDAC1 and induced mitochondrial dysfunction. PLAs using antibodies to VDAC1 and Aβ showed a 6-fold increase in the number of signals indicating complex formation between VDAC1 and Aβ when N2a cells were incubated with exosomes from 5xFAD serum as compared to those from wild type serum (Fig. 7a-c). PLA signals were clustered (arrows in Fig. 7b) consistent with mitochondrial clustering induced by Aβ-associated astrosomes. Mitochondrial damage was confirmed by up-regulation of the fission protein Drp-1 in mitochondria isolated from N2a cells incubated with 5xFAD serum exosomes (Fig. 7d). Figure 7e and f shows that in N2a cells and primary cultured neurons incubated with exosomes from 5xFAD mice or AD patient serum, PLA signals were colocalized with Tom-20, concurrent with mitochondrial clustering. These results confirm that astrosome-associated Aβ formed complexes with mitochondrial VDAC1 and leads to mitochondrial damage. PLA signals for VDAC1-Aβ complexes were also found in the vicinity of amyloid plaques of AD brain tissue, suggesting that VDAC1-Aβ complex formation contributes to AD pathology in vivo (Supplemental Fig. 4B).

**Fig. 8:**
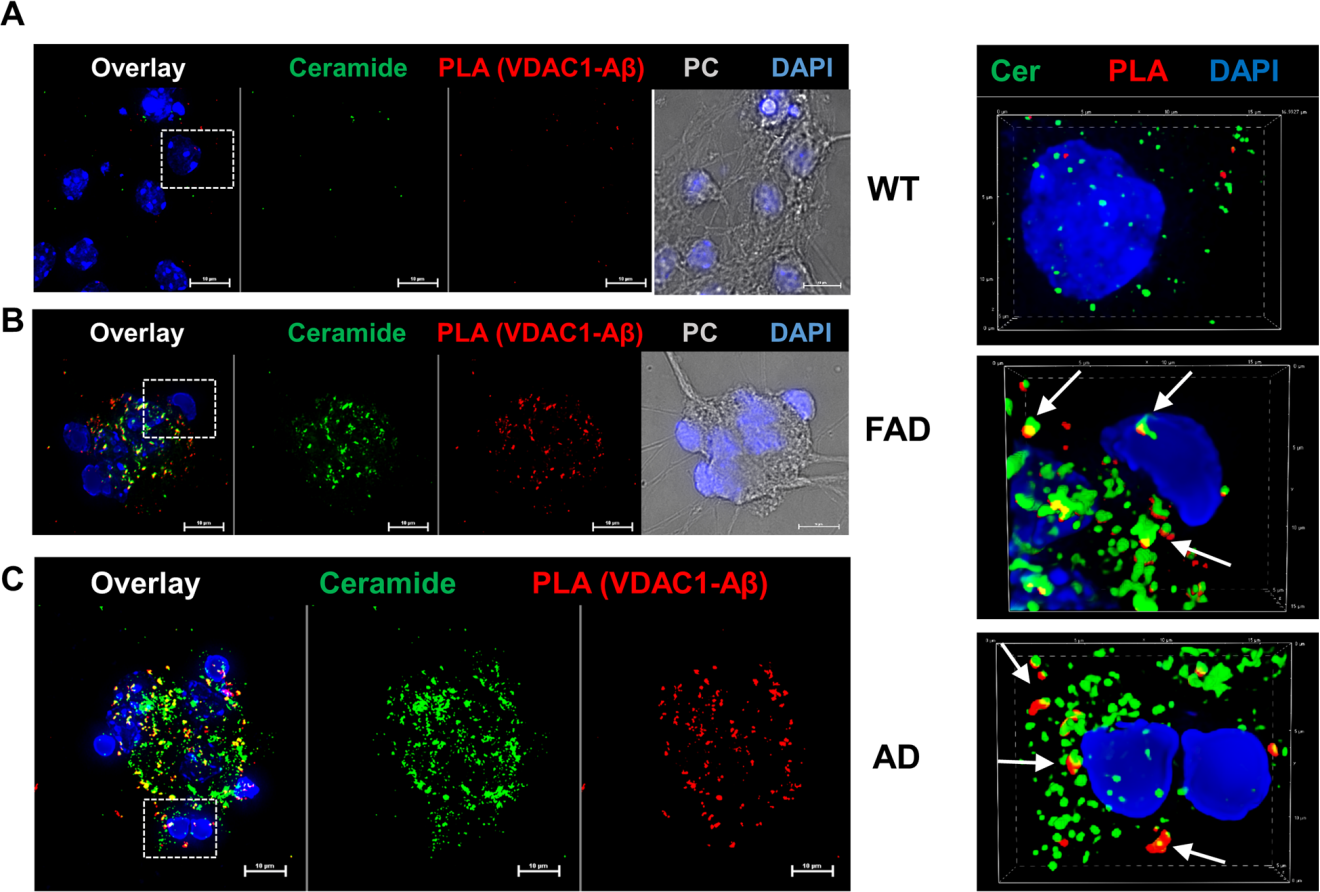
*Exosome-induced VDAC1-Aβ complex formation is associated with ceramide* Representative immunofluorescence images for PLA signals from VDAC1-Aβ complexes and ceramide in primary cultured neurons incubated with wild type mouse exosomes (**a**), 5xFAD mouse exosomes (**b**), or human AD patient serum-derived exosomes (**c**). Images in right panel are details at higher magnification (frames in left panel) with arrows pointing at PLA signals colocalized with ceramide

Since 5xFAD mouse and AD patient serum exosomes transported ceramide into cells (Figs. 3b and 4b) we tested if VDAC1-Aβ complex formation was colocalized with ceramide. Figure 8a-c shows that in primary cultured neurons incubated with 5xFAD serum exosomes (Fig. 8b) or AD patient exosomes (Fig. 8c), PLA signals for formation of complexes of VDAC1 with Aβ were colocalized with ceramide (arrows). Neurons incubated with wild type serum exosomes showed no or only a few PLA signals and they were not colocalized with ceramide (Fig. 8a). This result suggested that VDAC1-Aβ complex formation was associated with ceramide derived from exosomes.

**Fig. 9:**
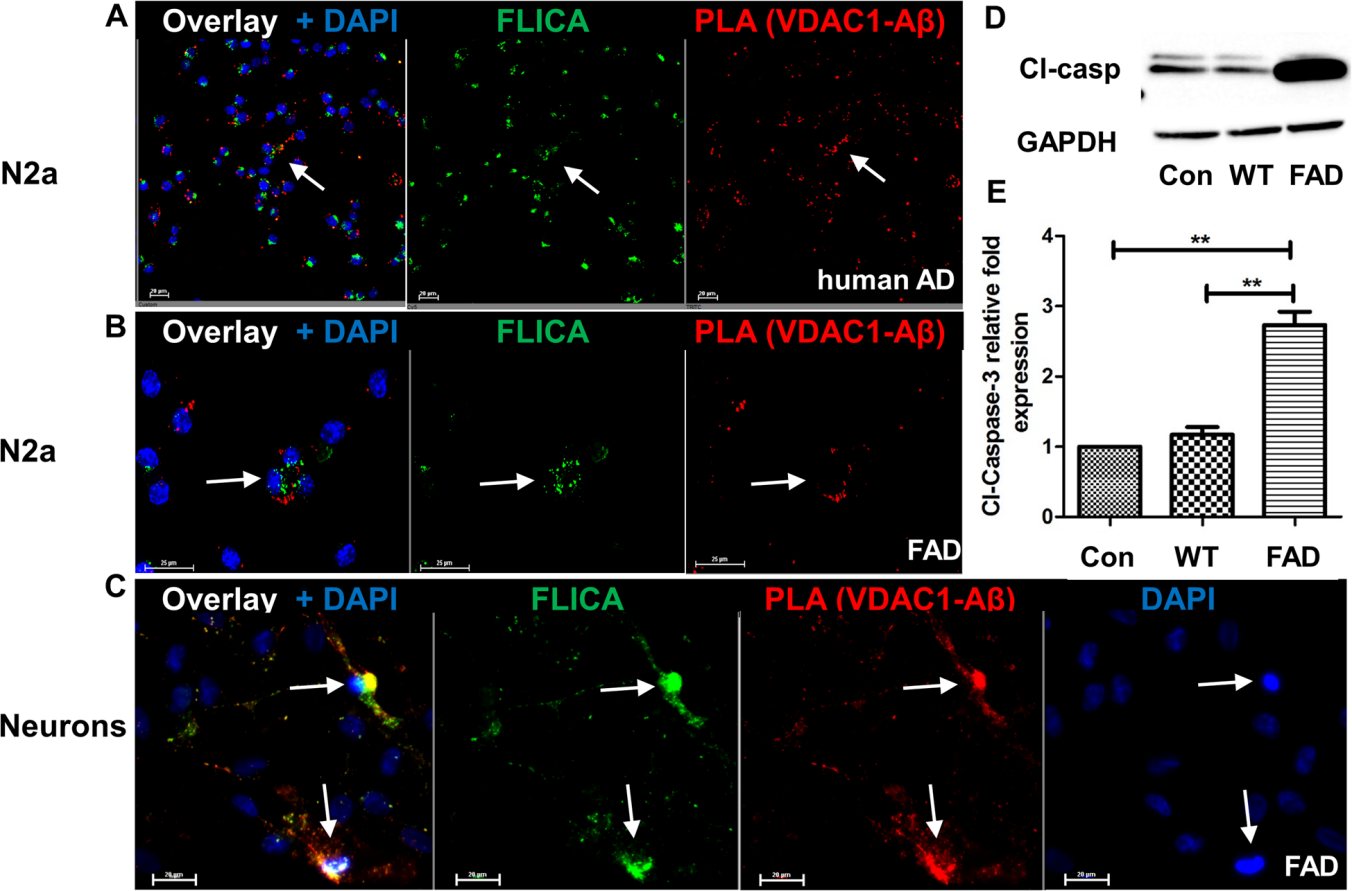
*5xFAD and human AD patient serum-derived exosomes trigger apoptosis in cells induced by interaction between mitochondrial VDAC1 and Aβ.* Representative immunofluorescence images of N2a cells incubated with (**a**) AD patient exosomes or (**b**) 5xFAD mouse serum-derived exosomes. FLICA assays were followed by PLAs for VDAC1-Aβ complex formation. Images show that cells with VDAC1-Aβ complexes undergo apoptosis (arrows). **c** Primary cultured neurons incubated with 5xFAD serum exosomes followed by FLICA assays and PLAs. Arrows indicate neurons colabeled for VDAC1-Aβ complexes and caspase 3 activation. These cells show pyknic nuclei (condensed DAPI labeling) indicative of apoptois. **d** Western blot with N2a cell lysate immunolabeled for cleaved caspase 3 using GAPDH as a reference protein. Blot is representative of three independent experiments. **e** Relative fold expression of cleaved caspase 3 normalized to GAPDH. One-way ANOVA followed by Tukey correction. *N* = 3. **p < 0.001

Next, we tested if exosome-mediated VDAC1-Aβ complex formation led to activation of caspase 3, a hallmark of neurotoxicity and apoptosis. Figure 9a and b shows that in N2a cells incubated with AD patient serum (Fig. 9a) or 5xFAD mouse serum (Fig. 9b) exosomes, PLA signals for VDAC1-Aβ complexes were colocalized with labeling for activation of caspases (FLICA assays), suggesting induction of apoptosis. Activation of caspases was confirmed by immunoblot analysis for cleaved caspase 3 (Fig. 9d and e). Since the Aβ content of 5xFAD serum exosomes was approximately 25 pg Aβ_42_/10^12^ exosomes (calculations based on ELISA data, not shown), and 10^4^ exosomes/cell were added to 10^5^ cells in 1 ml of medium, the apparent Aβ concentration was 5 fmoles/l, which is several orders of magnitude less than what is commonly used in Aβ neurotoxicity assays. VDAC1-Aβ complex formation concurrent with caspase 3 activation was confirmed with 5xFAD serum exosomes and primary cultured neurons (Fig. 9c), suggesting that association of Aβ to ceramide-enriched exosomes enhances Aβ neurotoxicity by inducing mitochondrial damage and caspase 3 activation.

**Fig. 10:**
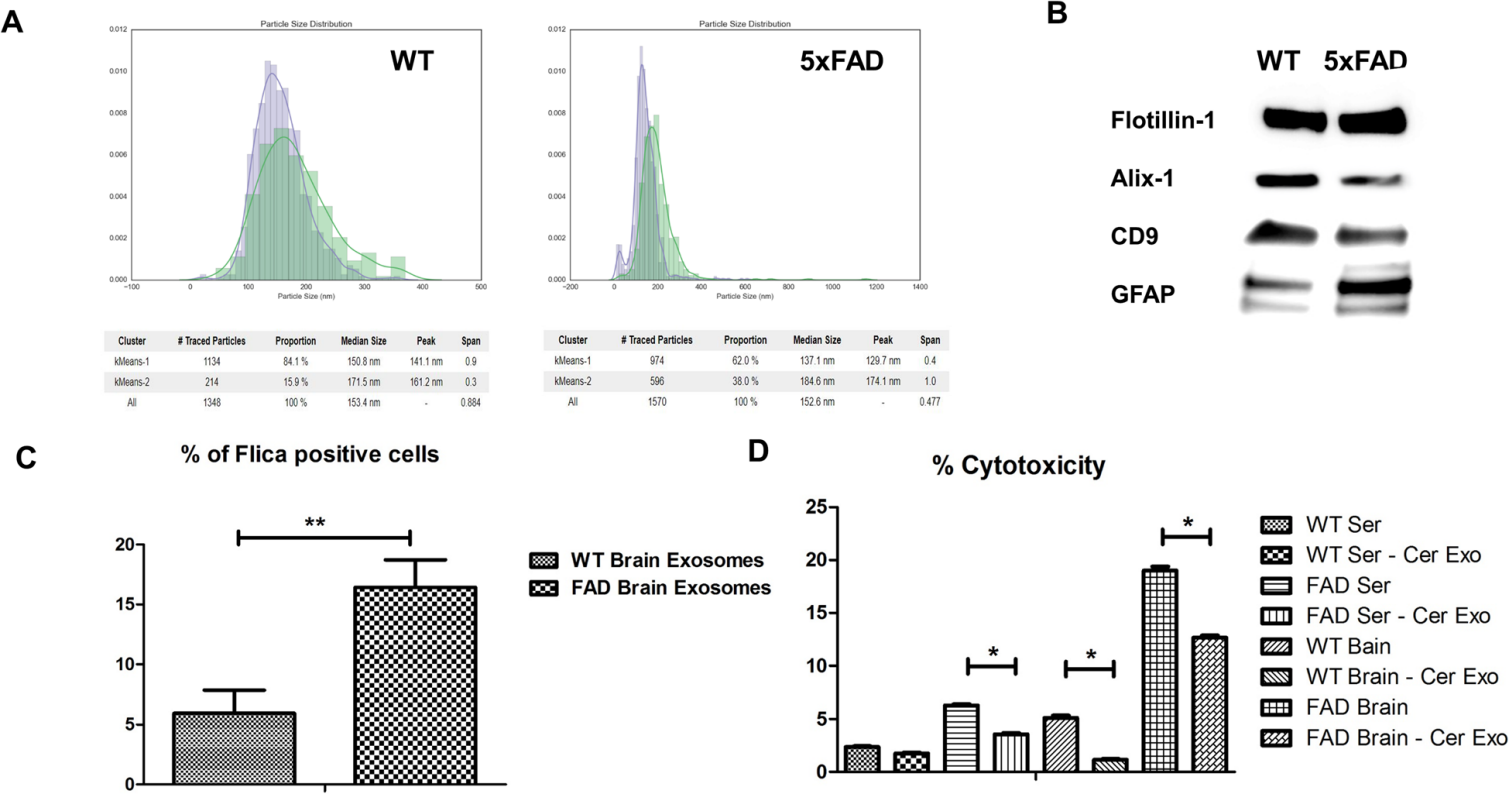
*Ceramide-enriched 5xFAD brain tissue and serum exosomes are neurotoxic.* (**a**) Cluster analysis of wild type (WT) and 5xFAD brain tissue-derived exosomes after Nano Particle Tracking analysis. *N* = 3 (**b**) Immunoblot of exosome markers flotillin-1, Alix-1, CD9, and GFAP, demonstrating higher amounts of GFAP in 5xFAD exosomes compared to WT exosomes. **c** FLICA assay shows 3-fold increased rate of apoptosis induction (cells with activated caspase 3) after incubation for 12 h of N2a cells with 5xFAD brain tissue exosomes compared to WT exosomes. *N* = 15, Student t-test with Welch’s correction *p* < 0.005. **d** Cytotoxicity (CyQuant) assay shows reduction of neurotoxicity after depletion of ceramide-enriched exosomes. *N* = 7, Multiple Student t-test with Welch’s correction *p* < 0.05

Finally, we compared neurotoxicity of 5xFAD serum exosomes with those from mouse brain tissue before and after removal of ceramide-enriched astrosomes using pull down with anti-ceramide antibody. Exosomes were isolated from brain tissue after perfusion with PBS to rule out any contamination with serum exosomes. Analyses using NTA showed that brain tissue-derived exosomes from wild type and 5xFAD mice were similar in number (1.5 × 10^9^ exosomes/mg wild type vs. 1.6 × 10^9^ exosomes/mg 5xFAD brain tissue) and size distribution (Fig. 10a). Normalized to exosome number, the wild type and 5xFAD exosomes contained comparable levels of exosome markers, however, GFAP levels were higher in 5xFAD exosomes indicative of a higher proportion of astrosomes in 5xFAD brain tissue (Fig. 10b).

Next, we tested if brain tissue-derived exosomes showed similar effects on neuronal apoptosis as serum exosomes. Results of the FLICA assay (Fig. 10c) show that induction of apoptosis by incubation with 5xFAD brain exosomes (0.5 × 10^4^ exosomes/cell) was about 3-fold higher than that with wild type brain exosomes, consistent with data obtained with exosomes from serum (Fig. 9). When testing cytotoxicity using a CyQuant assay we found that normalized on the same number of exosomes used per cell (10^4^ exosomes/cell) toxicity of exosomes from 5xFAD brain was 4-fold higher than that from wild type brain and 3.5-fold higher than that from 5xFAD serum exosomes. This result shows that the number of neurotoxic exosomes is highly elevated in 5xFAD brain and that serum exosomes represent a portion of brain exosomes with increased neurotoxicity. When we removed the portion of ceramide-enriched exosomes from 5xFAD and wild type brain tissue and serum, cytotoxicity dropped by half, demonstrating that enhancement of Aβ neurotoxicity is mediated by enrichment of Aβ-associated astrosomes with ceramide.

## Discussion

While accumulation of Aβ is a hallmark in human AD, its causal role in neurotoxicity and cognitive decline is persistently elusive. Other factors such as tau, bacterial or viral infection, insulin resistance, and neuroinflammation are invoked in AD, and yet none of these factors was proven critical in the onset of the disease or neurodegeneration [10, 34, 54]. Probably the most likely explanation for AD pathophysiology is a multifactorial cascade of events with any of these factors initiating or amplifying each other during the course of the disease. This multifactor hypothesis implies that each factor is necessary, but not sufficient to initiate AD or cause neurotoxicity. The idea that Aβ or tau require additional factors critical to mediate or enhance their neurotoxicity is not surprising. Many studies showed that Aβ and tau concentrations used to induce neuronal damage or death in vitro are often orders of magnitude higher than those found in vivo [7, 38, 40]. In addition, Aβ and tau concentrations or plaque and tangle size in vivo are often not correlated with the extent of neurodegeneration or cognitive decline [7]. Recently, extracellular vesicles (EVs), exosomes or microvesicles, were proposed as carrier for transport and uptake of Aβ and tau into neurons [20, 58]. However, it is not clear how this uptake may lead to neurodegeneration in AD. In this study, we show for the first time that exosomes are not only carrier for Aβ, but also sensitize neurons to Aβ toxicity.

Several studies showed that plasma or serum from AD mice and patients contains exosomes that are associated with Aβ, demonstrating that Aβ-associated exosomes crossed the blood brain barrier (BBB) [29, 41]. In AD patients, about 23% of these exosomes were found to be derived from astrocytes, while the remainder was from neurons. Association of astrocyte-derived exosomes (termed astrosomes in the current study) with Aβ_42_ was shown to be several-fold higher than that of neurons, suggesting that the primary source of Aβ-associated exosomes are astrocytes [30] It was not investigated, however, if Aβ-associated astrosomes were enriched with ceramide or taken up by cells and involved in the pathophysiology of AD. Using a method developed in our laboratory, lipid-mediated affinity chromatography (LIMAC) of vesicles with anti-ceramide antibody [3], we showed for the first time that astrosomes from serum were ceramide-enriched and associated with Aβ. Nanoparticle tracking analysis (Zetaview) of LIMAC fractions showed that 9.2% of serum-derived exosomes were Aβ-associated astrosomes, while the remainder (not bound by anti-ceramide antibody) were only weakly labeled for GFAP and likely of neuronal origin. The Aβ content in these vesicles was approximately 25 pg Aβ_42_/10^12^ exosomes, which corresponded to 250 μl of serum (5xFAD mice). Mass spectrometric (LC-MS/MS) analysis of serum-derived exosomes showed enrichment with ceramide species similar to those found in exosomes released by primary cultures of astrocytes in vitro [71]. This data prompted us to hypothesize that serum-derived astrosomes associated with Aβ by a mechanism similar to that previously published for in vitro-generated astrosomes.

In previous studies, we showed that anti-ceramide antibody prevented association of Aβ_42_ with in vitro-generated astrosomes [15]. We concluded that ceramide was critical for binding of Aβ_42_ to astrosomes, by either directly interacting with it or facilitating interaction of Aβ with other components of the vesicle membrane. In this study, we tested a novel concept using ceramide analogs originally developed in our laboratory to disrupt binding of Aβ to astrosomes [5]. The novel ceramide analog N-oleoyl serinol (S18) reduced the average diameter of exosomes from 5xFAD mice. This result suggests that S18-treated exosomes are less prone to aggregation, and probably, association of Aβ with exosomes is resolved.

To date, only a few studies addressed a potential function of Aβ-associated exosomes in AD. It was shown that exosomes can spread amyloid between neurons and that uptake of EVs isolated from the cerebrospinal fluid or plasma of AD patients impairs mitochondrial respiratory function and induces caspase activation [20, 58]. However, it was not shown that Aβ-associated astrosomes are ceramide-enriched, transported to mitochondria, and mediate Aβ-binding to a critical mitochondrial protein. Our previous studies suggested that Aβ-associated astrosomes induce nucleation of amyloid plaques and critically participate in neurodegeneration [17]. However, consistent with other reports showing that neurotoxicity is not directly correlated with plaque size, we hypothesized that Aβ-associated astrosomes mediate neurotoxicity by a mechanism distinct from plaque formation.

The results using exosomes from serum of 5xFAD mice and AD patients show that Aβ-associated astrosomes are transported to mitochondria. This is demonstrated by colabeling of Aβ and ceramide with Tom-20 in cells that are also positive for GFAP and flotillin 2 when exposed to serum exosomes from 5xFAD mice or AD patients, but not from wild type mice or healthy controls. These cells show mitochondrial damage as documented by clustering of mitochondria and increased levels of the mitochondrial fission protein Drp-1. Our data is consistent with that from previous studies reporting that the level of Drp-1 is elevated in AD brain and neurons exposed to Aβ in vitro [1, 42]. In our previous studies, we showed that Aβ exposure leads to mitochondrial malformation and dysregulation of VDAC1, the main ADP/ATP transporter in the outer mitochondrial membrane the level of which is elevated in AD [14, 37, 56, 59]. Our data is consistent with that from previous studies reporting that Aβ binds to VDAC1 and induces formation of a pro-apoptotic pore [68]. Using cortical protein lysates from AD patient and AD mouse model brains, it was shown by co-immunoprecipitation assay that Aβ binds to mitochondrial VDAC1 [43]. However, none of the previous studies investigated the effect of Aβ-associated exosomes on VDAC1 and its interaction with Aβ.

To test the role of Aβ-associated exosomes in the interaction of VDAC1 with Aβ we performed proximity ligation assays (PLAs) after exposure of N2a cells and neurons to exosomes from 5xFAD mice and AD patients as well as wild type mice and healthy controls. Our results show that exosomes from 5xFAD mouse or AD patient serum lead to PLA signals indicating formation of a complex between VDAC1 and Aβ. Therefore, we concluded that Aβ-associated exosomes induced or mediated complex formation between VDAC1 and Aβ. Currently, we are investigating the mechanism by which exosomes induce this complex formation.

**Fig. 11:**
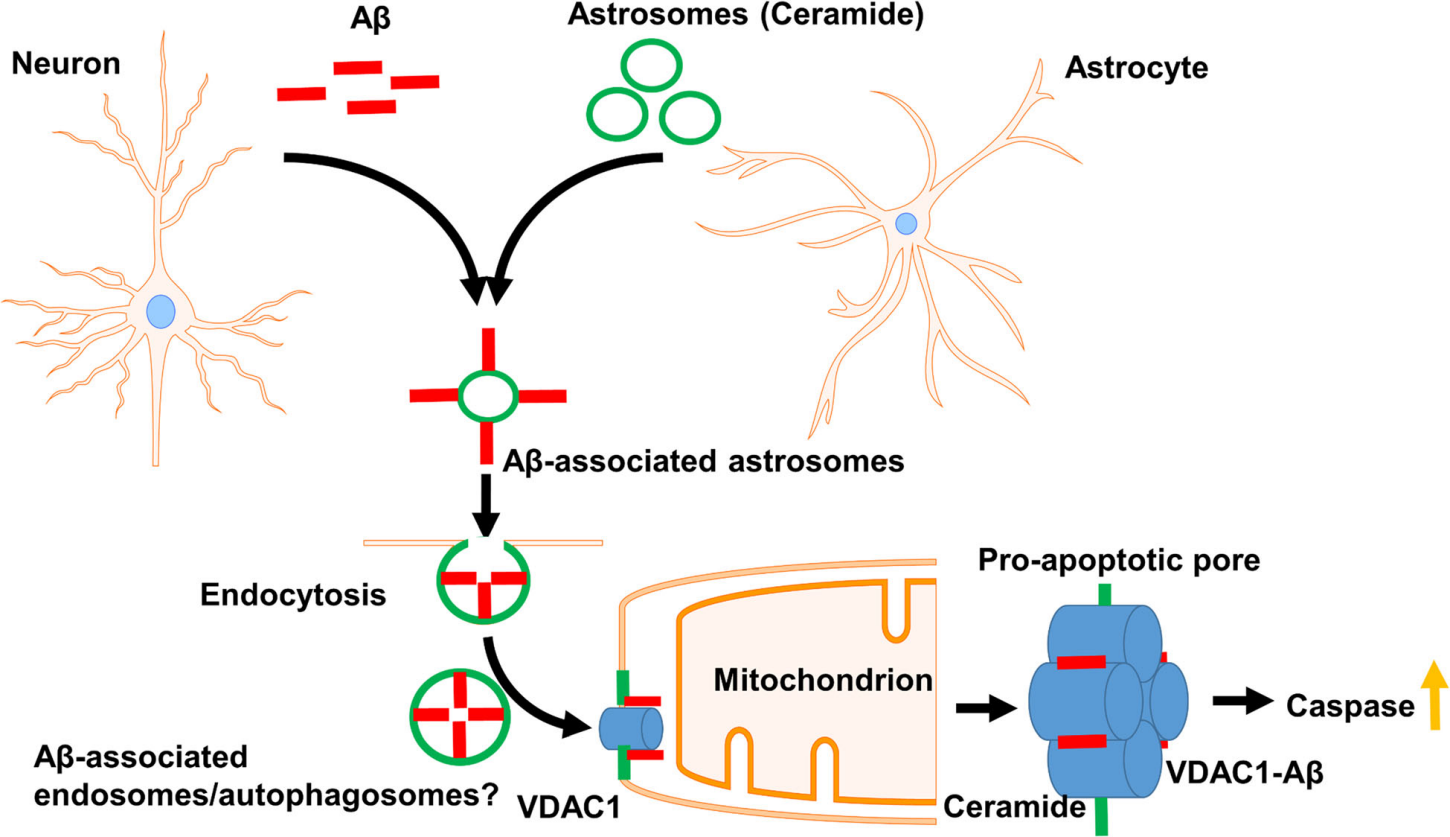
*Potential mechanism of neurotoxicity induced by Aβ-associated astrosomes.* Aβ secreted by neurons (red) binds to ceramide-enriched exosomes secreted by astrocytes (astrosomes, green). Aβ-associated astrosomes are endocytosed by neurons and transported to mitochondria. The vesicles fuse with the outer mitochondrial membrane and mediate binding of Aβ to VDAC1. A pro-apoptotic pore, probably associated with ceramide, is formed which leads to activation of caspases and induction of neuronal cell death

Figure 11 shows a model for endocytotic uptake and interaction with VDAC1 at mitochondria mediated by Aβ-associated astrosomes. Aβ-associated astrosomes may either be endocytosed as vesicles or first fuse with the plasma membrane. In both cases, Aβ (red in Fig. 11) remains associated with ceramide (green in Fig. 11), probably in the form of ceramide-rich platforms, a type of lipid rafts enriched with ceramide [4]. The persistent association with ceramide explains why Aβ and ceramide remain colabeled after uptake of Aβ-associated exosomes into N2a cells and neurons. Next, Aβ is shuttled to mitochondria, which is probably mediated by vesicular transport, either by Aβ-associated endosomes or other types of vesicular compartments such as aberrant autophagosomes [46, 48, 61, 73]. Finally, Aβ is imported into mitochondria to interact with VDAC1, which induces a pro-apoptotic pore that leads to release of cytochrome c and activation of caspases [68]. While interaction of Aβ with VDAC1 and formation of the pro-apoptotic pore was reported, the role of ceramide and exosomes in this process has not yet been investigated.

Ceramide was invoked in neuronal Aβ release and formation or secretion of exosomes from astrocytes [15–18]. Our studies showed that ceramide is instrumental for interaction of Aβ with astrosomes. In the novel mechanism depicted in Fig. 11, ceramide may critically participate in several steps of uptake, transport, and mitotoxicity of Aβ. Firstly, association of Aβ with ceramide in the astrosomal membrane may induce a specific Aβ isoform or aggregate promoting endocytosis. This hypothesis is consistent with our observation that a proportion of exosomes from serum of 5xFAD mice or AD patients form aggregates. Secondly, ceramide may critically participate in neuronal endocytosis and transport of Aβ to mitochondria. In numerous studies, it was shown that fluorescently labeled ceramide was taken up by endocytosis and then transported to specific compartments, mainly the Golgi apparatus [27, 31, 52]. Albeit the reason for Golgi accumulation is unclear, other studies support the idea that ceramide guides transport of endosomes to specific compartments, which may include those interacting with mitochondria [26, 72]. It should be noted that our previous studies showed that uptake of Aβ_42_ by glial cells is reduced by at least 50% when associated with exosomes, suggesting that uptake of Aβ-associated exosomes as observed in our current study is specific for neurons and potentially mediated by ceramide [17]. Thirdly, ceramide may participate in import of Aβ into mitochondria, e.g., by fusing Aβ-associated astrosomes to the outer mitochondrial membrane, and binding to VDAC1. Interaction of either ceramide or Aβ with VDAC1 was demonstrated by several studies from our and other laboratories [24, 37, 43, 44]. Alternatively, membranes closely associated with the outer mitochondrial membrane such as mitochondria-associated membranes (MAMs) may take part in the interaction of Aβ with VDAC1. Fourthly, ceramide may facilitate formation of a pro-apoptotic pore that is associated with the VDAC1-Aβ complex. VDAC1 oligomers as well as ceramide channels were reported to partake in pro-apoptotic pores at mitochondria [28, 66], however, the involvement of Aβ-associated and ceramide-enriched exosomes in formation of these pores was not discussed yet. Our observation that VDAC1-Aβ complexes are colocalized with ceramide at mitochondria suggests a novel mechanism by which association of VDAC1 with ceramide and Aβ induces or facilitates pro-apoptotic pore formation. Finally, since association of Aβ with exosomes is remarkably stable and persists during passage through the BBB into the blood stream, the proportion of Aβ-associated exosomes may participate in systemic distribution and potentially, reuptake of Aβ and its spreading throughout the brain.

Our data show that 5xFAD brain-derived and ceramide-enriched exosomes are neurotoxic and serum contains a proportion of these exosomes crossing the BBB. However, it is conceivable that these exosomes acquire additional toxic factors during their passage through the blood stream. Therefore, Aβ-associated exosomes in serum may not only be a biomarker and “window” to the brain, but actively participate in spreading AD pathology and contributing to Aβ neurotoxicity after reuptake into the brain. While we utilized serum-derived exosomes in our in vitro experiments to elucidate the proposed mechanism, studies are planned to further test the significance of ceramide-enriched exosomes for Aβ neurotoxcity in vivo. These studies will address the function of different ceramide species in neurotoxicity, particularly when comparing 5xFAD mice with AD patients and the in vivo significance of systemic distribution and reuptake of Aβ-associated astrosomes. Here we present for the first time experimental evidence for our hypothesis that ceramide and Aβ act synergistically to target VDAC1 and induce caspase activation, ultimately leading to neuronal malfunction and apoptosis. Therefore, Aβ-associated astrosomes assisting in Aβ uptake, transport, and mitotoxicity are a novel key factor in sensitizing neurons to Aβ and a potential pharmacological target to prevent neurodegeneration in AD.

Current pharmacological approaches exclusively aim at interfering with ceramide generation using inhibitors for enzymes in ceramide metabolism [8, 39, 60]. The most prominent example is GW4869, an inhibitor for neutral sphingomyelinase 2 (nSMase2) we have shown to reduce plaque formation and improve cognition in male 5xFAD mice [15]. We previously reported that the nSMase2- deficient 5XFAD mice (fro;5XFAD) showed a reduced number of brain exosomes, ceramide levels, glial activation, total Aβ_42_ and plaque burden, and improved recognition in a fear-conditioned learning task [17]. In future studies, we will specifically address the function of astrocyte-derived exosomes in Aβ neurotoxicity by including mice with astrocyte-specific deletion of nSMase2 as well as knockouts of individual ceramide synthases.

While enzyme inhibitors are promising as lead compounds interfering with ceramide metabolism in AD, alternative pharmacological approaches targeting ceramide but not depending on enzyme inhibition may offer additional benefits. About 20 years ago, our laboratory designed and synthesized novel ceramide analogs of the β-hydroxy alkylamine type, particularly N-oleoyl serinol [6] (S18) that do not inhibit ceramide generation, but interfere with binding of ceramide to its protein interaction partners such as atypical protein kinase C λ/ξ [6]. These analogs were shown to be non-toxic to normal cells, but induce apoptosis in cancer cells [5]. We hypothesized that novel ceramide analogs may also interfere with binding of Aβ to ceramide in astrosomes, thereby providing a novel therapeutic approach preventing astrosome-mediated spreading and uptake of Aβ, and sensitization of neurons to Aβ. Our data with S18 obliterating exosome aggregates in 5xFAD serum support this hypothesis, which will also be investigated in our future research. In summary, our data show for the first time that astrosomes sensitize neurons to Aβ and suggest that interfering with binding of Aβ to astrosomes using novel ceramide analogs may provide a novel therapeutic strategy for treating AD.

## Conclusions

The exact mechanism of amyloid beta (Aβ) peptide neurotoxicity in Alzheimer’s disease (AD) is not known. Extracellular vesicles (EVs), specifically exosomes were recently found to bind and spread Aβ. In this study, we showed that exosomes secreted by astrocytes (astrosomes) associated with Aβ and were taken up by neurons and transported to mitochondria. Aβ-associated astrosomes were enriched with the sphingolipid ceramide that is suggested to mediate binding of Aβ to voltage-dependent anion channel 1 (VDAC1), the main ADP/ATP transporter in the outer mitochondrial membrane. The VDAC1-Aβ complex is known to form an oligomeric pro-apoptotic pore. Hence, association of Aβ with astrosomes targeting mitochondria in neurons is a novel mechanism to enhance Aβ neurotoxicity by inducing apoptosis. Our data show that this mechanism induced neuronal apoptosis at a concentration (5 femtomolar) that is several orders of magnitudes lower than that achieved with Aβ without exosomes. Neurotoxic Aβ-associated astrosomes were isolated from brain tissue and serum indicating that they cross the blood-brain barrier. Neurotoxicity of brain and serum astrosomes is critically dependent on ceramide, suggesting that disruption of Aβ association or other effects of astrosomes with ceramide analogs offers a new therapeutic approach to AD.

## Supplementary information

**Additional file 1.**

## Data Availability

All data generated or analysed during this study are included in this published article [and its supplementary information files].
