# Supplementary material for: Association of Aβ with ceramide-enriched astrosomes mediates Aβ neurotoxicity
Source: Acta Neuropathol Commun. 2020 Apr 28;8:60. doi: 10.1186/s40478-020-00931-8 (PMC7189561; doi:10.1186/s40478-020-00931-8)
Supplement: Supplementary file 1 — Additional file 1. [file 40478_2020_931_MOESM1_ESM.pdf]

Supplemental Figures

Supplemental Figure 1

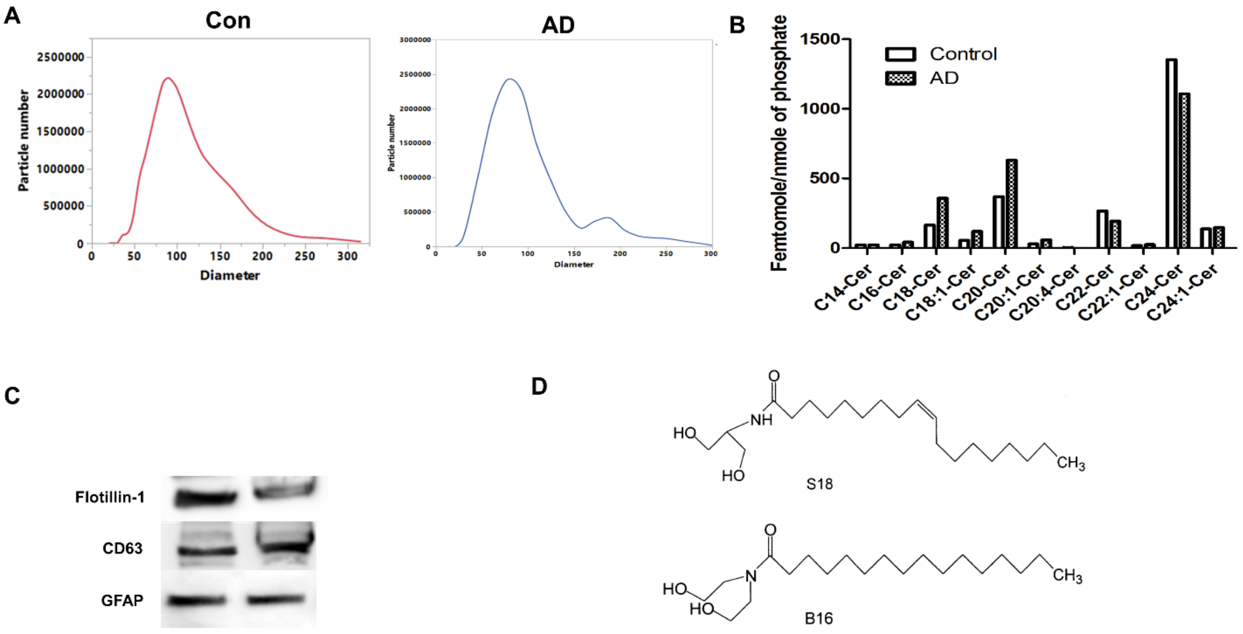

Serum-derived exosomes from AD patients are enriched with ceramide. (A) Size distribution (Zetaview NTA analysis) of human serum exosomes. (**B**) Ceramide species profile using lipid mass spectrometry (LC-MS/MS) of AD patient serum-derived exosomes normalized to phosphate content. **(C)** Immunoblot for exosome markers CD63 and Flotillin-1 showing equal protein expression levels of GFAP in AD and healthy control individuals. **(D)** Structures of ceramide analogs S18 and B16.

Supplemental Figure 2

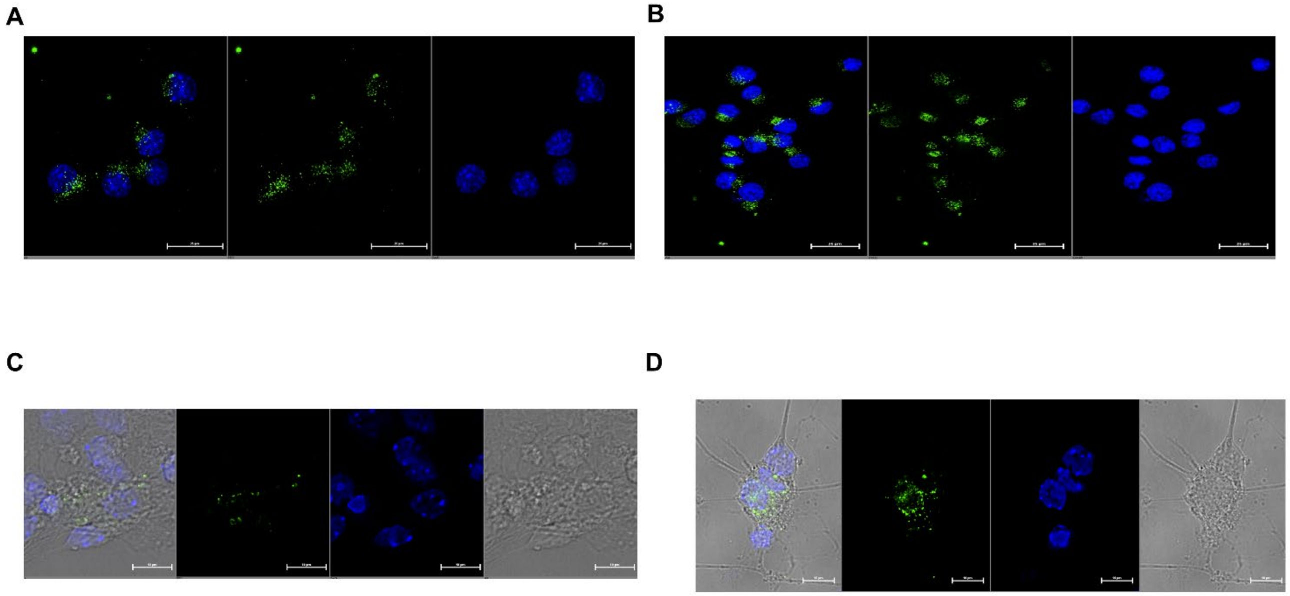

Serum derived exosomes from WT and 5xFAD mice are taken up by N2a cells. Representative fluorescence microscopy images of PKH67-labeled exosomes from wild type (**A**) and 5xFAD (**B**) mice showing their uptake by N2a cells and primary cultured neurons (**C**, wild type; **D**, 5xFAD exosomes).

Supplemental Figure 3

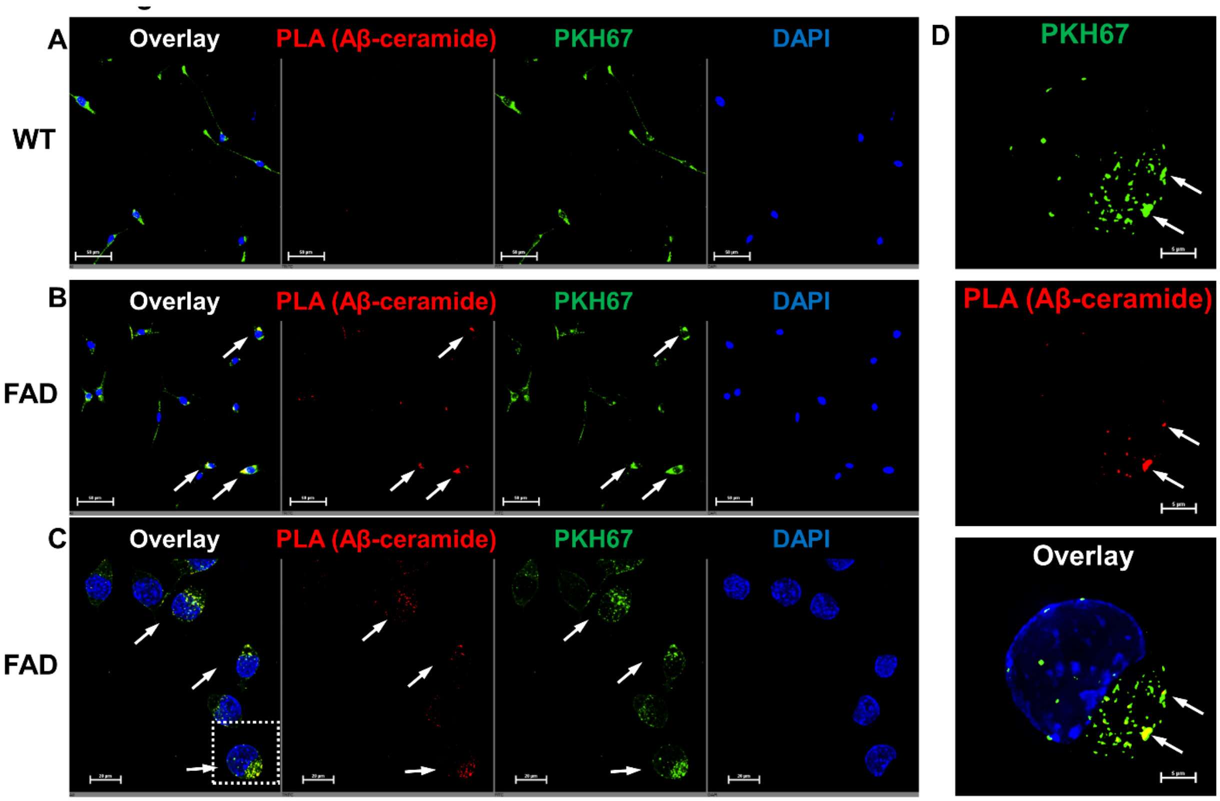

5xFAD exosomes retained complex formation between Aβ and ceramide after uptake into N2a cells. Either wild type (**A**) or 5xFAD (**B, C, D**) serum-derived exosomes were labeled with PKH67 dye and then then used for incubation of N2a cells. PLA shows complex formation between Aβ and ceramide only with 5xFAD exosomes. C is similar to B at higher magnification. D is detail (frame) from C.

Supplemental Figure 4

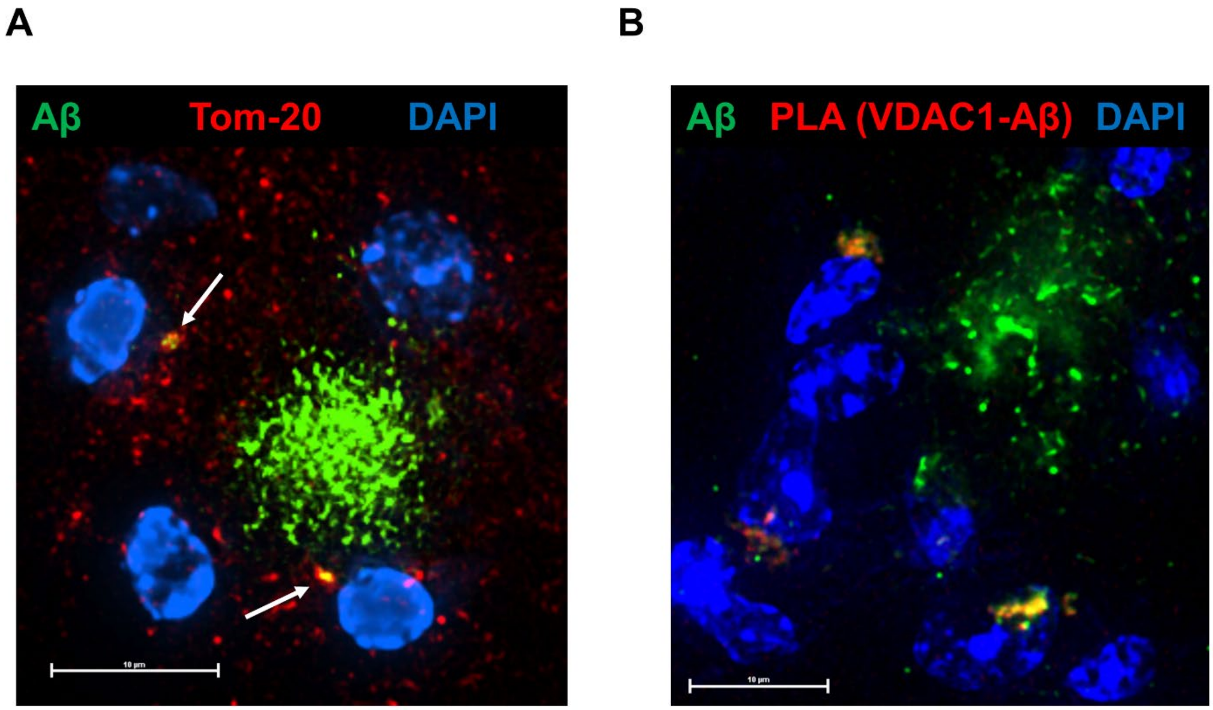

Interaction between Aβ and mitochondrial via VDAC1 in human brain. (**A**) Representative flouresence image of human brain section showing colocalization of Aβ with mitochondrial Tom-20 aroung amyloid plaque (arrows). (**B**) PLA using antibodies against Aβ and mitochondrial VDAC1 showing complex formation in cells surrounding amyloid plaque.
